# Antagonistic action of Siah2 and Pard3/JamC to promote germinal zone exit of differentiated cerebellar granule neurons by modulating Ntn1 signaling via Dcc

**DOI:** 10.21203/rs.3.rs-1819367/v1

**Published:** 2024-09-25

**Authors:** Christophe Laumonnerie, Maleelo Shamambo, Daniel R. Stabley, Tommy L. Lewis, Niraj Trivedi, Danielle Howell, David J Solecki

**Affiliations:** 1- Neuronal Cell Biology Division, Department of Developmental Neurobiology, St. Jude Children’s Research Hospital, 262 Danny Thomas Place, Memphis, TN 38104; 2- Aging & Metabolism Program, Oklahoma Medical Research Foundation, 825 NE 13th Street, Oklahoma City, OK 73104

**Keywords:** Germinal Zone, Polarity, Netrin1, Dcc, Pard3, JamC, Coincidence Detection

## Abstract

Exiting a germinal zone (GZ) initiates a cascade of events that promote neuronal maturation and circuit assembly. Developing neurons and their progenitors must interpret various niche signals—such as morphogens, guidance molecules, extracellular matrix components, and adhesive cues—to navigate this region. How differentiating neurons integrate and adapt to multiple cell-extrinsic niche cues with their cell-intrinsic machinery in exiting a GZ is unknown. We establish cooperation between cell polarity-regulated adhesion and Netrin-1 (Ntn-1) signaling comprises a coincidence detection circuit repelling maturing neurons from their GZ. In this circuit, the Partitioning defective 3 (Pard3) polarity protein and Junctional adhesion molecule-C (JamC) adhesion protein promote, while the Seven in absentia 2 (Siah2) ubiquitin ligase inhibits, Deleted in colorectal cancer (Dcc) receptor surface recruitment to gate differentiation linked repulsion to GZ Ntn-1. These results demonstrate cell polarity as a central integrator of adhesive- and guidance cues cooperating to spur GZ exit.

## Introduction

During nervous system development, sequential events ensure the formation of the neuronal circuitry required for proper cognitive function. At the cellular level, maturing neurons navigate multifaceted tissue environments by interpreting cell-extrinsic factors that cooperate with cell-intrinsic programs to bring about the stereotypical progression of cells from neuronal progenitors to mature neurons with proper identity, location, and connectivity [1]. Flawed execution of these genetically encoded but environmentally regulated programs has drastic consequences for brain morphogenesis and circuit function, and it ultimately leads to a wide range of pediatric neurological disorders [2]. There is compelling evidence that extrinsic factors, such as guidance molecules and intrinsic programs, such as those involving neuronal polarity signaling or cytoskeletal organization, are important for germinal zone (GZ) exit, migration, and synaptogenesis in circuit formation. However, understanding how cell biological programs like neuronal polarity signaling coordinate with extrinsic guidance signals remains a significant challenge due to a reductionist focus on how molecular players function in isolation and a lack of cell biological or imaging approaches to tackle this issue mechanistically. This study addresses this challenge by revealing how cell polarity signaling, cell adhesion, and the sensing of guidance molecules work in unison at the cell biological level to control when neuronal progenitor cells or newly differentiated neurons leave their germinal niche.

Granule neurons from the mouse cerebellar cortex have long been a system of choice for studying how neuronal progenitor replication in proliferative niches transitions to differentiation of neurons that migrate out of their GZ to a final laminar position [3–5]. In the outer external granule layer (oEGL), granule neuron progenitors (GNPs) exhibit a low-polarity morphology. As they differentiate, newborn cerebellar granule neurons (CGNs) progressively extend a leading process parallel to the surface of the cerebellar cortex and start migrating along that axis in the inner external granule layer (iEGL). Eventually, CGNs extend a perpendicular process, and the soma starts migrating radially on the surface of Bergmann glia fibers to reach the inner granule layer (IGL), leaving the axon behind in the molecular layer. This model is ideal for understanding how disparate cell biological processes act in unison. Recent work has shown that transcriptional and post-transcriptional regulation of polarity complex protein availability is critical for GZ exit. GNPs express the E3 ubiquitin ligase Siah2, which targets Pard3 for degradation [6]. Additionally, hypoxic pressure in the GZ and expression of the transcription factor Zeb1 transcriptionally repress Pard6α and Pard3 mRNA expression, thus maintaining cells in the EGL [7, 8]. As CGNs differentiate, the loss of transcriptional and post-translational repression, marked by diminished expression of Siah2 and Zeb1, leads to the expression of polarity proteins. This change enables one neurite to specialize as a leading process, promoting the recruitment of JamC to the cell surface [6]. Consequently, JamC facilitates adhesion to radial glial fibers, providing a substrate for migration through the molecular layer [9, 10]. Despite the well-developed role of this polarity-dependent adhesion system in GZ exit, we still do not understand how this critical pathway is linked to classical guidance molecules that could functionally align cellular asymmetry to external reference points as newborn neurons take up their positions in the cerebellar circuit.

A large array of extrinsic cues have been identified as making short-range and long-range contributions to the cell cycle regulation of GNPs and the migration of CGNs [5]. This myriad of signals raises the question of how a given neuron can filter out only those cues relevant to its behavior at a given time. We hypothesized that cellular polarization and polarity complex proteins could modulate the strength of extrinsic guidance signals by regulating the access of transmembrane receptors to the membrane surface. To test this idea, we investigated the function of the guidance molecule Ntn1 in guiding CGNs out of the GZ. Ntn1 signaling is transduced by different transmembrane receptors of the immunoglobulin superfamily, like the deleted in Dcc and Uncoordinated protein 5 (Unc5) transmembrane proteins, and is involved in axonal guidance and neuronal migration [11–14]. Based on the combination of receptors expressed by the cell, the signal can mediate attraction or repulsion behavior [14]. Although Ntn1 is not known to guide the migration of GNPs from the upper rhombic lip in the cerebellar system, postnatal cerebellar cortex explants showed that Ntn1 repelled parallel fiber extensions; however, the function of neither Dcc nor Unc5 receptors were formally assessed at the time [15]. In addition, the mutant model for another Ntn1 receptor, Unc5c, has been described as possessing a smaller cerebellum and folia, ectopic cerebellar cells in the midbrain, and abnormal postnatal cerebellar migration [11].

In this study, we used the GZ exit of differentiating CGNs in the cerebellum as a model to study how extracellular guidance cue–receptor pairs, such as Dcc and Ntn1, a cellular polarity complex, and an adhesion protein can intersect, creating a coincidence detection circuit that modulates cellular migration. Our results reveal a localized source of Ntn1 in the EGL, where GNPs reside, and Unc5c upregulation promotes GZ exit, specifically of newly differentiated CGNs. Levels of Dcc receptors were found to be critical for tuning the sensitivity of the response. Using FACS-sorted populations of GNPs and CGNs, we show that differentiated neurons migrate away from a source of Ntn1, whereas progenitors show no preferential migration orientation. These observations led us to investigate how the access of transmembrane receptors to the membrane surface was regulated. Like Pard3, Dcc has been previously reported to be targeted for degradation by the proteasome by Siah proteins [16–18]. Here we show that Siah2 promotes GZ occupancy by decreasing the amount of Dcc receptors at the membrane. Using super-resolution microscopy, we provide evidence that Dcc receptors reside near Pard3 and JamC at the membrane surface; additionally, a proximity labelling assay (PLA) shows that JamC, Pard3, and Dcc form a protein complex. We demonstrate that Pard3 and JamC functions are essential to a proper Ntn1-mediated migration phenotype in an ex vivo assay. Necessity–sufficiency testing revealed that Pard3 promotes Dcc receptor clustering in response to netrin-1, whereas JamC controls the basal levels of Dcc at the surface. Thus, Pard3 and JamC regulate the sensitivity of newly differentiated CGNs to the netrin gradient by adjusting the number of Dcc receptors recruited to the cell surface. This new paradigm for cooperativity between disparate cell biological processes illustrates that coincidence detection between JamC and polarity-dependent adhesion and sensitivity to a repulsive Ntn1 work in tandem to facilitate GZ exit, with the polarity pathway concentrating Dcc at cell surface locations by stabilizing and/or promoting transmembrane receptor exocytosis. Siah2 ubiquitin ligase activity antagonizes these complexes via ubiquitin-based turnover, thus favoring progenitor GZ occupancy.

## Results

**Figure 1.**
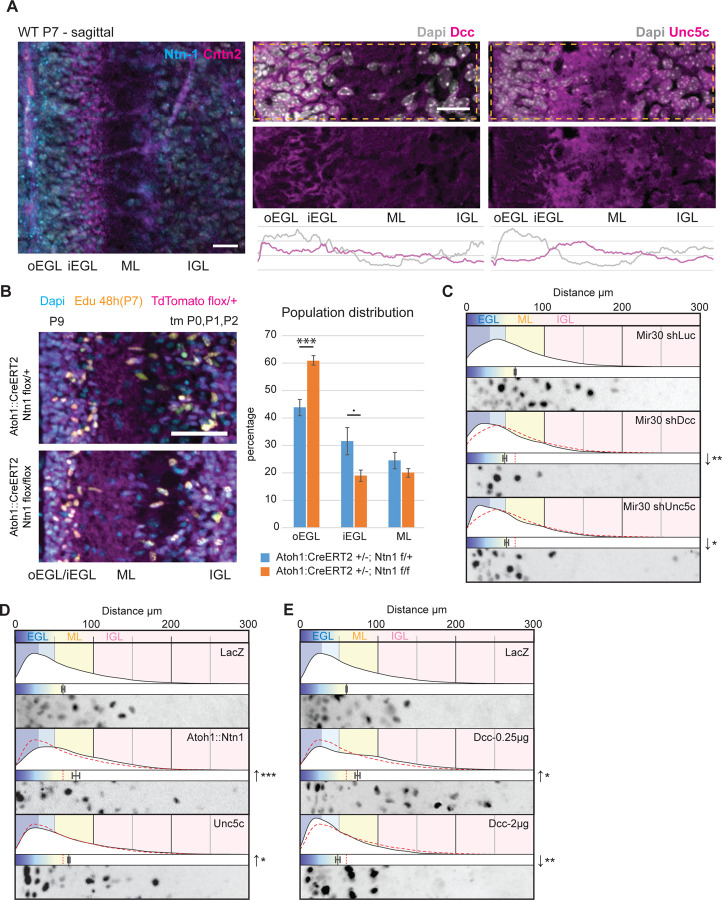
(A) Immunohistochemical staining of sagittal cryo-sections of wild-type and Atoh1-GFP mouse cerebella at P7. From left to right, the sections were stained used antibodies against Ntn1 (cyan) and Cntn2 (magenta), Dcc (magenta), and Unc5c (magenta), and were counterstained with Dapi (white). Dashed-line boxes highlight regions used for average intensity plot over the radial axis. (B) Sagittal cryo-sections through the cerebella of P9 Rosa::TdTomato^fl/+^; Atoh1::CreERT2^+/−^; Ntn1^flox/wt^ or Ntn1^flox/flox^ animals after three doses of tamoxifen (tm) at P0, P1, and P2 and a single injection of Edu at P7. Sections were stained for Edu, and the radial distance from the edge of the Edu^+^ nucleus was measured and plotted. oEGL: 0–30 µm; iEGL: 30–50 µm; ML: 50–100 µm. (C)–(E). Results of ex vivo slice culture assays under different conditions. In each case, the top curve shows the entire distribution of the radial distances of H2B-positive electroporated nuclei from the edge of the slice in replicates. Below this is a plot of the average radial distance from the edge among replicates, and below this is a micrograph representative of the nuclear distribution in that assay. All are displayed on the same scale, representing a distance from 0 to 300 µm. In addition to H2B-Cherry, the following constructs were electroporated: in (C), Mir30 shLuc (control), Mir30 shDcc, and Mir30 shUnc5c; in (D), LacZ (control), Atoh1::Ntn1, and Unc5c; in (E), LacZ (control), Dcc at 0.25 µg, and Dcc at 2 µg. Each respective control is represented by a red dashed-line in the distribution plot. Scale bars in (A) and (B) represent 20 µm and 50 µm, respectively. Abbreviations: oEGL, outer external granule layer; iEGL, inner external granule layer; EGL, external granule layer; ML, molecular layer; IGL, internal granule layer. In (B) through (E), the error bars represent the SEM. Statistics: *p ≤ 0.05; **p ≤ 0.01, ***p ≤ 0.005, as assessed by a Student *t*-test in (B) and an ANOVA followed by a Dunnett post hoc test in (C-E) against the respective controls. See also Table S1.

Ntn1 signaling has been implicated in guiding early aspects of cerebellar morphogenesis, such as repulsion of CGN axons and the positioning of the boundary of the forming EGL [11, 15], making it an excellent guidance molecule model for assessing cooperativity with polarity proteins. While their mRNA expression was analyzed previously, we re-assessed the expression of Ntn1 and its receptors Dcc and Unc5c at the protein level in postnatal day 7 (P7) mice. Immunohistochemical staining revealed that Ntn1 is present in the oEGL complementary to Contactin2 (Cntn2) CGN marker expression in the iEGL. In contrast, immunohistochemical staining for Ntn1 receptors showed that Dcc and Unc5c are expressed strongly in the iEGL. Whereas Dcc expression is also in the oEGL, especially close to the Pia, Unc5c appears more restricted to the iEGL and ML (Figure 1A). Unc5c’s restricted expression pattern was surprising as previous analyses of its mRNA expression showed expression in all cerebellar layers. These expression patterns suggest that Ntn1 and its receptors are well-positioned to affect CGN GZ exit and/or layer occupancy decisions at the transition zone between iEGL and ML.

We next assessed whether Ntn1 function affected GZ exit in the developing cerebellum in vivo by using a Cre-inducible system to generate Atoh1::CreERT2/Ntn1^lox/lox^; Rosa::TdTomato^lox/wt^ mice, as this has not been done in previous studies. In vivo GZ pulse-chase analysis was conducted in animals to whom tamoxifen was administered daily from P0 to P3, using one dose of 5-ethynyl-2´-deoxyuridine (EdU) injected at P7. The EdU GZ pulse labels the proliferative GNPs of the oEGL in the cerebellar cortex when injected at P7. The position of Edu/TdTomato-positive cells in tissues collected at later stages of development can be used as a proxy to analyze the GZ exit and migration of the CGN progeny of GNPs in vivo. At P9, the average distance from the cerebellar surface of Edu/TdTomato-positive nuclei, which is a measure of GZ exit and subsequent migration, was lower in Atoh1::CreERT2/Ntn1^lox/lox^; Rosa::TdTomato^lox/wt^ mice than in their Atoh1::CreERT2/Ntn1^lox/WT^; Rosa::TdTomato^lox/wt^ littermates, showing that deleting Ntn1 from the EGL results in the inhibition of GZ exit (Figure 1B). Given that Ntn1 has been previously described as an oncogene, limiting apoptosis induced by its receptors Dcc and Unc5s [19], we assessed proliferation and apoptosis status. In antibody staining experiments on wild-type and mutant animals, we denoted EGL subdivisions using Cntn2 immunostaining: iEGL (Cntn2 positive) and oEGL (Cntn2 negative), along with the actively cycling neuronal progenitor marker Ki67 and the apoptosis marker Cleaved-Caspase3. The results showed no differences between WT and knock-down animals at this developmental stage (Figure S1A, B). Additionally, dissociated CGN cultures supplemented with Ntn1 demonstrated a significant reduction in the proportion of progenitor cells after 48 hours of culture (Figure S1C), indicating that Ntn1 does not directly maintain proliferation. Therefore, the increased EGL occupancy of EdU-labeled neurons observed in the Ntn1 knockdown of the Atoh1 lineage (Figure 1B) is likely due to a migratory defect.

Given the expression of Dcc and Unc5c in differentiated CGNs, we next assessed the function of these receptors in GZ exit by using the *ex vivo* slice culture assay developed in our laboratory, as the functional role of these receptors has not been assessed in the CGN lineage. Silencing of either Unc5c or Dcc via Mir30 shRNA expression significantly reduced the average radial migration distance from the pial surface after 48 h in culture and increased the proportion of cells that remained in the EGL (Figure 1C). These results are consistent with studies showing that Dcc/Unc5 heterodimers mediate chemorepulsion from a source of Nnt1 [14], buttressing our finding that Ntn1 loss of function led to enhanced GZ occupancy. Interestingly, the average migration speed was not affected by Dcc and Unc5c silencing in dissociated CGN cultures (Figure S1D), showing that the loss of GZ exit in silenced cells is not due to defective cell motility.

Given the necessity of Ntn1, Dcc, and Unc5c for CGNs to take up positions outside the EGL, we next tested whether they were sufficient to induce GZ exit. Expressing Ntn1 in *ex vivo* slices by using the Atoh1 regulatory sequence to restrict its expression to the oEGL increased the average radial distance of electroporated nuclei from the pial surface and reduced EGL occupancy (Figure 1D). Elevated expression of Unc5c, which is expressed only as CGNs differentiate, resulted in a similar precocious GZ exit phenotype (Figure 1D). Expression of Dcc exhibited a concentration-specific phenotype: a mild elevation of Dcc expression promoted GZ exit, whereas at higher concentrations, cells remained in the GZ (Figure 1E). This phenotypic transition was gradual (Figure S1E) and, based on the literature, could reflect the fact that increasing the number of Dcc receptors at the cell surface favors Dcc homodimer formation and attraction to a source of Ntn1 [14, 20], whereas when the number of Dcc receptors is low, they may cooperate with endogenous Unc5, promoting repulsion. These results show that Dcc expression and the regulation of its availability at the membrane surface appear to be critical to modulating the GZ exit response to Ntn1 in the oEGL.

**Figure 2.**
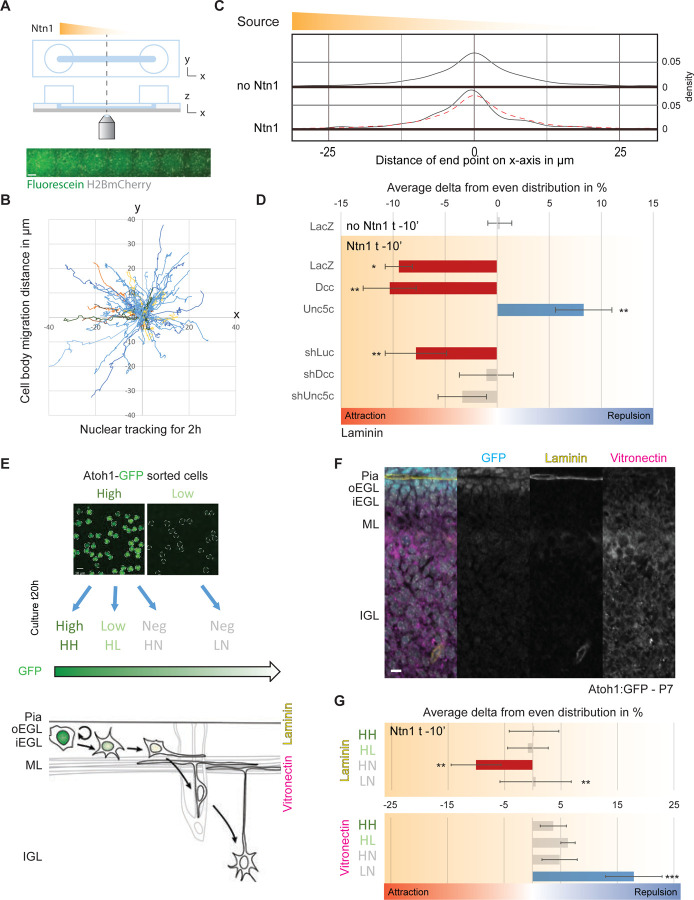
(A) Schematic representing the channel microslides used to assess migratory behavior in response to the unilateral addition of Ntn1 from time-lapse imaging. The panel below shows tiled pictures of the dissociated cerebellar granule neurons nucleofected with H2B-mCherry (white) within a fluorescein gradient (green) created in the middle area of the channel microslide. (B) Example of manual tracking of nuclear motion during the 2-h time-lapse imaging session, here without the addition of Ntn1. Each track is centered at t0, and motions are displayed as the relative displacement from the origin. The x-axis is aligned with the channel length, as depicted in (A). (C) Graph representing the density distribution of the endpoint displacements on the x-axis across all of the tracked migration in the “no Ntn1” control (upper graph and red dashed-line in lower graph) (n = 338) and with the unilateral addition of Ntn1 as depicted by the “source” bar at top (n = 379). The gradient was set 10 min before the start of the 2-h time-lapse imaging session. (D) Chart representing the average variation across replicates in the endpoint nuclear displacement on the x-axis from an even probability of 50%:50%, with negative values representing an attraction to the source of the gradient (no Ntn1 or Ntn1) (in red when statistically significant) and positive values representing repulsion (in blue when statistically significant). Dissociated cerebellar granule neurons were plated on laminin-coated channel microslides 24 h before tracking began, and the gradient was set 10 min before the start of image acquisition. Cells were nucleofected with H2B-mCherry, GPI-pHluorin, and the following: LacZ (control), Dcc, Unc5c, shLuc (control), shDcc, or shUnc5c. (E) Top. Plated cells after dissociation from cerebella of Atoh1-GFP mice and sorting by FACS into high-GFP-intensity and low-GFP-intensity populations, showing Atoh1-GFP signal (green). Bottom. Schematic representation of the cerebellar cortical layers of a P7 Atoh1-GFP mouse, showing the decrease in GFP intensity during the differentiation process and the migration of a GNP from the oEGL to the IGL and the corresponding switch of the extracellular matrix from laminin-rich to vitronectin-rich. (F) Immunohistochemical staining of a sagittal cryo-section of Atoh1-GFP mouse cerebellum at P7 with antibodies against GFP (cyan), laminin (yellow), and vitronectin (magenta). (G) Chart organized like (D) representing the bias in the nuclear migration of different populations of FACS-sorted Atoh1-GFP mouse cerebellar neurons plated on channel microslides coated with either laminin or vitronectin after unilateral addition of Ntn1 10 min before the start of the 2-h time-lapse imaging. Scale bars in (A), (E), and (F) represent 100, 10, and 10 µm respectively. Abbreviations: oEGL, outer external granule layer; iEGL, inner external granule layer; EGL, external granule layer; ML, molecular layer; IGL, internal granule layer. In (D) and (G), the error bars represent the SEM. Statistics: *p ≤ 0.05, **p ≤ 0.01, ***p ≤ 0.005, as assessed by chi-square test against an even probability of 50%:50%. See also Table S1.

To assess whether Ntn1 signaling directly affected CGN migration direction, we used an in vitro system to control the source of this guidance cue. Channel microslides can set and hold a gradient for several hours, enable different substrate coatings to be placed on the glass base of the slide, and are optically compatible with time-lapse microscopy. Dissociated cerebellar neurons were nucleofected and seeded into the channel of a microslide and incubated for 24 h. Ten minutes before the start of an imaging experiment, the medium in the channel was replaced, and that in the left half of the slide was replaced with medium containing 200 ng/mL of recombinant Ntn1 or its equivalent in volume in 1× PBS as a control. Fluorescein was also added to enable visualization of the gradient status over time (Figure 2A). The motion of H2b-mCherry–labeled nuclei in the middle of the channel was assayed via 2 h of live-cell time-lapse microscopy followed by computational tracking of nuclei to determine the cell response to an oriented Ntn1 source (Figure 2B). For simplicity, we expressed the tracking results as variations from a 50%:50% distribution in the x-axis of the slide, as there were no specific alterations in the relative endpoint displacement in the gradient y axis. Although the endpoint distribution of migration on the x-axis showed no preferential direction in control cells, exposure to the Ntn1 gradient resulted in an attractive shift toward the source of Ntn1 (Figure 2C). A chi-square statistical test revealed that the pattern of attraction observed with Ntn1 was significantly different from a 50%:50% distribution (Figure 2D). To validate the dependency of migration on Ntn1 signaling, we nucleofected constructs to modify the expression levels of the Ntn1 receptors. On laminin-coated slides, a gain of function of Dcc maintained the attraction phenotype observed in controls, whereas the gain of function of Unc5c reversed the phenotype to a repulsive one, with cells migrating away from the source of Ntn1 (Figure 2D). Mir30-based shRNA knockdown of both Dcc and Unc5c resulted in the loss of responsiveness to the source of Ntn1 (Figure 2D). We should note that shUnc5c cells trended towards attraction, but experimental variability rendered the p-value of the Chi-squared statistical test above the 0.05 level. However, this result is still consistent with a loss of GZ exit for Unc5c deficient CGNs in the cerebellar slice assay. Overall, these results confirm that the Ntn1 receptors Dcc and Unc5c are essential to reorienting somal migration of dissociated CGNs in response to a source of Ntn1.

Given the differentiation-specific expression of Dcc and Unc5c, we were curious as to whether GNPs and CGNs at various differentiation stages had distinct responses to Ntn1. We took advantage of the knock-in mouse line in which EGFP is fused in-frame with the Atoh1 transcription factor explicitly expressed in GNPs (Figure 2E). Atoh1-EGFP fluorescence had two uses in our experiment: 1) Atoh1-EGFP fluorescence could be used to sort dissociated EGL cells into GNPs (Atoh1-high, Pax6^+^) and CGNs (Atoh1-low, Pax6^+^) (Figure S2A), with Atoh1-negative cells from the initial sort being in majority Pax2^+^ interneurons (Figure S2B); and 2) the intensity of Atoh1-EGFP in time-lapse imaging assays reported the differentiation status of the purified CGN lineage cells in real-time. In the Ntn1 migration assay, Atoh1-high and Atoh1-low populations were plated on microslides 24 h before time-lapse imaging their response. We also coated microslides with extracellular matrix components that are enriched near the oEGL (laminin) or iEGL (vitronectin) to better recapitulate conditions near the EGL niche (Figure 2F). Our time-lapse imaging revealed that the Atoh1-high population maintained a high level of Atoh1-GFP (true GNPs; designated HH cells), transitioned to a low level of Atoh1-GFP (intermediate GNPS; designated HL cells), or lost Atoh1-GFP altogether (recently differentiated CGNs; designated HN cells) (Figure 2E). Cells from the “low” sorted population remained negative for Atoh1-GFP and represented more mature CGNs (designated LN cells in Figure 2E). On both substrates, the HH and HL populations of Atoh1-GFP–positive cells showed no significant directional bias when migrating in the Ntn1 gradient (Figure 2G). On laminin, the HN population of newly differentiated CGNs migrated significantly toward the source of Ntn1 but showed no preference on vitronectin (Figure 2G). Conversely, the LN population of more mature CGNs migrated away from the Ntn1 source on vitronectin, which mimics the extracellular matrix components in the iEGL and the molecular layer. The behavior of the LN population on laminin was heterogeneous. A comparison to an even distribution with a chi-square test (Figure 2G) revealed that some cells had an attraction response (Figure S2C, magenta arrowhead). In contrast, others had a strong repulsion response (Figure S2C, cyan arrowhead), indicating that HL cells exposed to laminin could have a slower transition from attraction to repulsion. It is worth noting that vitronectin promotes CGN differentiation [21, 22], whereas laminin, in some contexts, maintains GNPs and CGNs in an immature state [22, 23]. Collectively, our in vivo, ex vivo, and microslide experiments show that the level of Dcc protein is critical for the regulation of GZ exit by Ntn1 signaling (Figure 1E) and that GNPs and CGNs display distinct responses to the presence of Ntn1 in the EGL GZ that differ according to their maturation status (Figure 2G).

**Figure 3.**
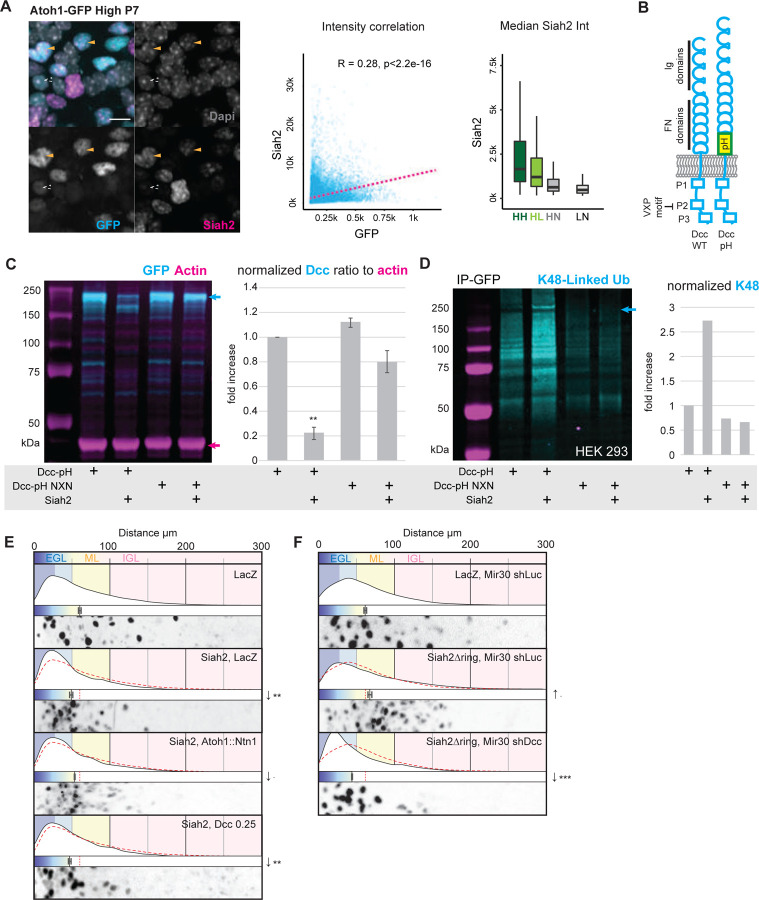
(A) (Left) Immunocytochemistry staining of the “High” and “Low” (not shown) population of Atoh1-GFP FACS-sorted CGNs from P7 mouse cerebellum and cultured for 24h, against GFP (Cyan) and Siah2 (Magenta), counterstained with Dapi (Gray). Cell nuclei were segmented with Stardist for quantification. The first graph shows the significant Pearson correlation between GFP and Siah2 signal intensity in all segmented cell bodies (“High” and “Low”). Boxplot shows the distribution of Siah2 intensity for the 4 populations previously defined in Figure 2 of granule neurons based on GFP intensity from the “High” sort: HH (->“High”), HL (->“Low”) and HN (->“Neg” (negative)) and the “Low” sort: LN (->”Neg”). (B) Schematic representing the different domains of Dcc, a single-pass transmembrane receptor for Ntn1 with 4 Immunoglobulin-like (Ig) domains and 6 fibronectin-like (FN) domains on its extracellular side and three intracellular domains, P1, P2, and P3. The schematic indicates the location of the canonical Siah2 degron motif VXP within P2. Additionally, on the right, a schematic representation of the modified Dcc-pH showing the insertion of a pH-sensitive version of GFP between the transmembrane domain and the FN domains. (C) Western blot of lysates of HEK293T cells after lipofection with Dcc-pHluorin (Dcc-pH) (Ctrl), Dcc-pHluorin NXN mutant (Dcc-pH NXN), and Siah2. The blot was immunostained with antibodies against GFP (cyan) and actin (magenta). Graph depicts the Dcc to actin ratio normalized to Ctrl. (D) Western blot of HEK 293 cells lysates immunoprecipitated with an antibody against GFP after the cells were lipofected with Dcc-pHluorin (Dcc-pH), Dcc-pHluorin NXN mutant (Dcc-pH NXN), and Siah2. Cells were treated with MG132 for 6 h before lysis. The blot was immunostained with an antibody against K48-linked ubiquitin (cyan). The arrow points to the lack of K48 ubiquitin– positive staining with the Dcc-pH NXN construct. Graph depicts the K48-Linked ubiquitin intensity, at the arrow level, normalized to Ctrl. (E) and(F). Results of ex vivo slice culture assays under different conditions. In each case, the top curve shows the entire distribution of the radial distances of H2B-positive electroporated nuclei from the edge of the slice in replicates. Below this is a plot of the average radial distance from the edge among replicates, and below this is a micrograph representative of the nuclear distribution in that assay. All are displayed on the same scale, representing a distance from 0 to 300 µm. In addition to H2B-Cherry, the following constructs were electroporated: in (E), LacZ (control), Siah2 and LacZ, Siah2 and Atoh1::Ntn1, and Siah2 and Dcc at 0.25µg; in (F), LacZ and Mir30 shLuc (control), Siah2Δring and Mir30 shLuc, and Siah2Δring and Mir30 shDcc. Scale bar in (A) represents 50 µm. Abbreviations: EGL, external granule layer; ML, molecular layer; IGL, internal granule layer. In (C), (E) and (F), the error bars represent the SEM. Statistics: p ≤ 0.1, *p ≤ 0.05, **p ≤ 0.01, ***p ≤ 0.005, as assessed by a Student *t*-test in (C) and an ANOVA followed by a Dunnett post hoc test against the respective controls. See also Table S1.

We were intrigued by the differentiation-specific expression of Dcc in CGNs and that Dcc expression levels modulated the response to Ntn1 in the *ex vivo* cerebellar slice assay. Accordingly, we assessed how Dcc levels are controlled during CGN differentiation. Previous work from our laboratory has shown that Siah2, a Ring-domain E3 ubiquitin ligase, is strongly expressed in GNPs and that it regulates GZ occupancy by targeting for degradation key proteins of the polarity complex, primary cilia, and hypoxia pathway [6, 8, 22, 23]. Furthermore, we show that Siah2 expression decreases in tandem with Atoh1-GFP fluorescent signal as CGNs differentiate and migrate deeper into the cerebellum (Figure S3A). Indeed, the sorted cells we used for our channel microslide experiments (e.g., HH, HL, HN, and LN cells defined in the last section) significantly vary in their relative Siah2 expression level in addition to their behavior in an Ntn1 gradient (Figure 3A). While some synergy exists between Siah2 activity and the mitogenic factor Sonic hedgehog (Shh) that prevents GNPs from differentiating [23], Shh does not directly drive mRNA Siah2 expression in dissociated CGNs culture (Figure S3B), suggesting a post-transcriptional reduction in CGN differentiation.

The Dcc protein carries the canonical degron motif Pro-X-Ala-X-Val-X-Pro, which is recognized by Siah proteins within its intracellular P2 domain [24] (Figure 3B). This motif is well conserved in organisms ranging from *Xenopus* to humans (Figure S3C) and has been shown to promote specific proteasomal degradation of Dcc by Siah proteins [17]. To confirm the specificity of Siah2-mediated degradation of Dcc, we co-expressed Siah2 with Dcc fused to pHluorin, a pH-sensitive fluorescent protein (Dcc-pH, (Figure 3B) in HEK293T cells. We also co-expressed Siah2 with a mutant form of Dcc-pH, Dcc-pH NXN, in which the last valine and proline of the Siah degron motif are replaced by two arginines, thereby abrogating Siah degradation [24] (Figure 3C). Whereas Siah2 decreased the level of Dcc-pH protein, that of Dcc-pH NXN remained unaffected by co-expression with Siah2. Furthermore, immunoprecipitation with an antibody against GFP showed that the K48-linked ubiquitin signal was more abundant when Dcc-pH was co-expressed with Siah2 but was absent when Dcc-pH NXN was co-expressed with Siah2 (Figure 3D). Therefore, Siah2 degradation of Dcc is a likely mechanism for restricting Dcc expression to CGNs, as these cells lack Siah2 expression.

Next, we assessed whether Siah2 regulation of Dcc protein levels was relevant to CGN GZ exit by conducting epistasis analysis in our ex vivo cerebellar slice and microslide assays. Elevated Siah2 expression inhibited enhanced GZ exit that was elicited by Ntn1 and Dcc gain of function, showing that Siah2 antagonizes Dcc function in GZ exit (Figure 3E). Indeed, Dcc silencing inhibited the boost in GZ exit that is elicited by overexpression of a dominant-negative form of Siah2 lacking the Ring domain (Siah2ΔRING) [6, 16], showing that the additional GZ exit that accompanies Siah2 inhibition relies on Dcc function, as would be expected from the Siah2–Dcc antagonism (Figure 3F). These results suggest that Siah2 expression in GNPs actively hampers GZ exit by targeting Dcc for degradation.

Our previous work showed that Siah2 regulates GZ exit by acting as a negative regulator of partitioning defective (Pard) cell polarity pathways that promote GZ exit. Given that Dcc has been described as a neuronal polarity inducer [25] that, according to our findings, regulates GZ exit and is also negatively regulated by Siah2, we were curious whether there was a relation between the Pard complex and Dcc. Pard3 also contains the Siah2 degron motif, and the proteasomal degradation of the latter prevents Pard3 from recruiting JamC to the cell membrane surface, resulting in CGNs accumulating in the GZ [6].

**Figure 4.**
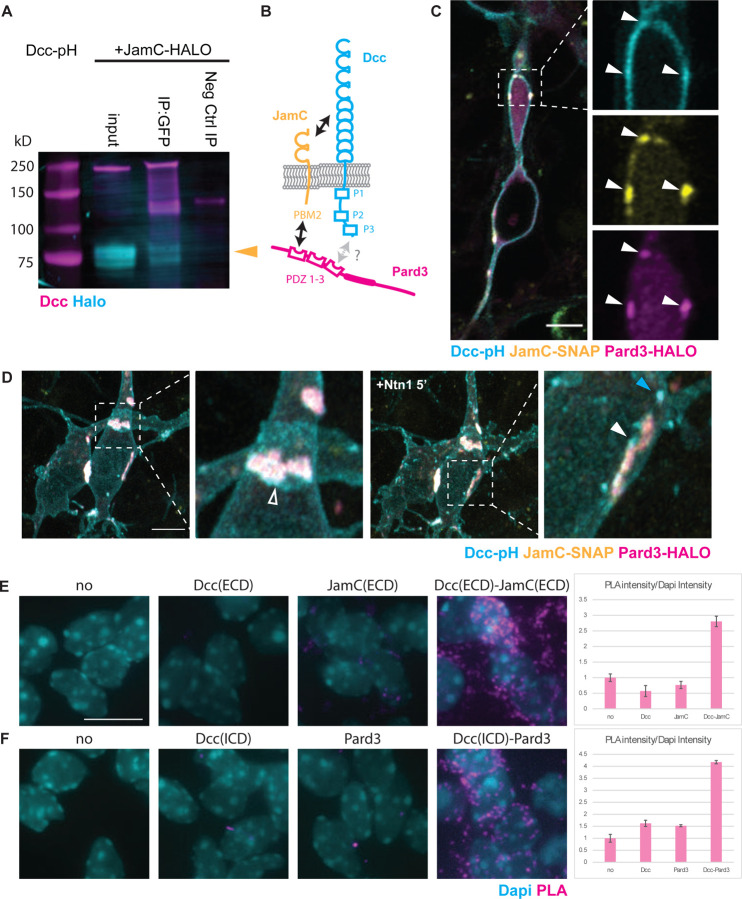
(A) Western blot of lysates of HEK293T cells after lipofection with Dcc-pHluorin (JamC-pH), and JamC-HALO. Cell lysates were immunoprecipitated with an antibody against GFP (IP: GFP) or a negative-control rabbit IgG (Neg Ctrl IP). The blots were immunostained with antibodies against Dcc and HALO-tag. Yellow arrowhead highlights the expected band size for JamC-HALO. (B) Schematic representing the predicted interactions between Dcc, JamC, and the polarity protein Pard3. Pard3 recruits JamC to the cell surface via its PDZ domain PDZ 1, which interacts with the Class 2 PDZ binding motif at the C-terminal of JamC. JamC interacts with the Dcc extracellular domain. Additionally, the PDZ 3 domain of Pard3 is predicted to interact with a Class 1 PDZ binding motif like the one present on the Dcc intracellular domain (X-S/T-X-ϕCOOH) [50, 51]. (C)–(D) Airyscan confocal live imaging of CGNs nucleofected with Dcc-pHluorin (cyan), JamC-SNAP (yellow), and Halo-Pard3 (magenta) constructs. Phluorin and the SNAP dye used here are both pH sensitive, emitting fluorescence only if exposed to a neutral pH. Thus, they show only proteins currently at the cell membrane surface. (C) A single focal plane 200 nm in thickness, showing overlap of the Dcc signal with the signals for JamC and Pard3 in the proximal dilation of a CGN (indicated by white arrowheads in the magnification). (D) Maximum projection of two CGNs forming an adhesion before and after the addition of Ntn1 to the medium. At the point of contact between the two cells, the Dcc signal overlapped the Pard3 and JamC signals (see magnifications). Additionally, Dcc accumulated also at the edge of the adhesion without overlapping with Pard3 or JamC (hollow arrowhead). Five minutes after the addition of Ntn1 at 200ng/L, the number of bright Dcc clusters (blue arrowhead) at the membrane surface increased and some newly formed clusters were recruited to the periphery of the JamC/Pard3/Dcc-positive adhesion (white arrowhead). (E-F) Proximity Labelling Assay (PLA) using Duolink^™^ fluorescence protocol on fixed dissociated granule neurons plated on laminin and cultured for 24h, using 2 pairs of primary antibodies: Rabbit against Dcc extracellular domain (ECD) and a Goat against JamC ECD (E), and Mouse against Dcc intracellular domain (ICD) and a Rabbit against Pard3 (F). Duolink^™^ staining with no primary, only one or both primaries were compared. Bar graphs represent the ratio of PLA staining intensity (Gray) against Dapi (Cyan) intensity normalized to the negative control with no primary antibody for all conditions. Scale bars in (C) and (D) represent 5 µm, and in (E) represents 10 µm.

JamC is a single pass transmembrane protein containing 2 Ig domains that can form homophilic interactions in Cis and Trans [6]. While Dcc requires specific ligand binding, it has also been recently described in forming cis and trans homophilic interactions [26]. To test whether Dcc interacted in a complex with JamC, we expressed these proteins in HEK293T cells and showed that JamC-Halo was present in the pull-down when Dcc-pHluorin was immunoprecipitated with an anti-GFP antibody (Figure 4A). The JamC–Dcc interaction required the Dcc extracellular domain, as JamC was able to pull-down the extracellular domain of Dcc but not the intracellular domain alone (Figure S4A). Dcc-pH could also pull down Pard3-Halo (Figure S4B), but we were less confident about this interaction because Pard3-Halo showed periodic binding to the immunoprecipitation beads. Immunocytochemical staining of dissociated CGNs plated on laminin revealed areas in which the Dcc signal overlapped with the signal for Pard3 or JamC or both (Figure S4C). We designed fusion proteins carrying reporters to enable us to image all three proteins in live neurons with super-resolution. CGNs expressing Dcc-pH, JamC-SNAP (stained with LAMPshade magenta [27]), and Halo-Pard3 were imaged with an Airyscan microscope, which has approximately 100-nm XY resolution. The pHluorin fluorescent protein fused to Dcc and the LAMPshade magenta dye that stained JamC-SNAP shares a pH-dependent fluorescence such that we imaged only the population of Dcc and JamC that was exocytosed to the extracellular face of the plasma membrane, which has a neutral pH when compared to the interior of a vesicle. When expressed together, the Dcc signal overlapped with that of JamC at the cell surface in locations where cells formed the point of contact with other cells in large, bright adhesion plaques (Figures 4C–4D; Figure S4D). Whereas the JamC signal appeared stable, Dcc was more dynamic in the middle of the JamC domain and coalesced at the edge of some JamC-labeled adhesions (Movie S1). The DCC signal sometimes coincided with the underlying Pard3 signal in fast-moving retrograde or stable Dcc clusters (Figures 4C–4D; Figure S4E; Movie S2). When Dcc, JamC, and Pard3 were co-expressed together, Pard3 accumulated below the large plaque of JamC that also overlapped with a brighter Dcc signal (Figure 4C, white arrowhead). It is important to note that Dcc did not localize exclusively to JamC/Pard3 structures and was distributed as a diffuse signal or in a clustered (brighter) structure at the membrane surface. Bright Dcc clusters often accumulated around a JamC/Pard3 bright adhesion between two cells (Figure 4D, hollow arrowhead). After Ntn1 was added to the medium, Dcc aggregated in clusters (Figure 4D, cyan arrowhead), some of which were recruited to JamC/Pard3 plaques (Figure 4D, hollow arrowhead).

As Dcc coincidence with JamC and Pard3 appears to involve a subset of the total Dcc pool within the cell, we assessed their proximity in situ using the Duolink^®^ proximity labelling assay [28]. We used a pair of antibodies targeting the respective extracellular domains (ECD) of Dcc and JamC and a pair of antibodies targeting the intracellular domain (ICD) of Dcc and Pard3. While negative control only showed minimal background, the proximity labeling assay was positive between the Dcc and JamC ECD, as well as Dcc ICD and Pard3, demonstrating a ~40 nm proximity between the polarity proteins and Dcc guidance receptor [28]. Regardless, the proximity of Dcc, JamC, and Pard3 in CGNs and the interactions detected in immunoprecipitation experiments suggest that Dcc forms a complex with polarity-related proteins such as JamC and Pard3.

**Figure 5.**
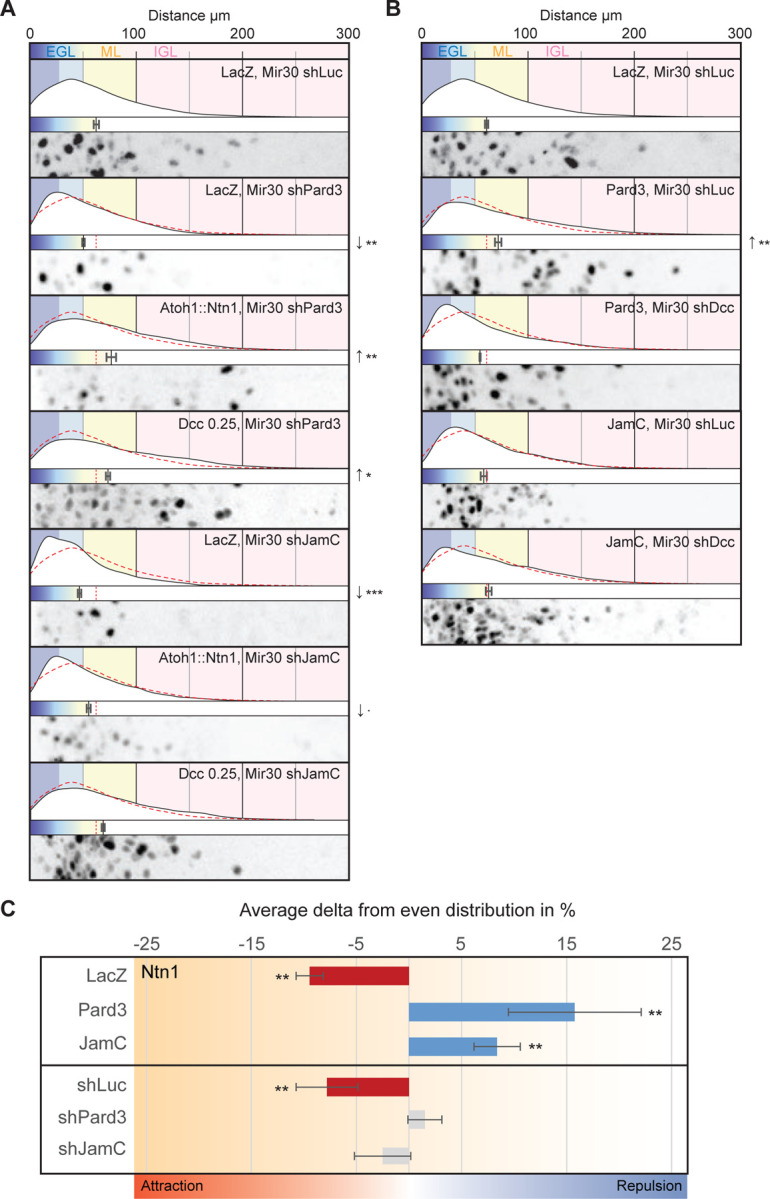
(A) and (B) Results of ex vivo slice culture assays under different conditions. In each case, the top curve shows the entire distribution of the radial distances of H2B-positive electroporated nuclei from the edge of the slice in replicates. Below this is a plot of the average radial distance from the edge among replicates, and below this is a micrograph representative of the nuclear distribution after 48 h in culture. All are displayed on the same scale, representing a distance from 0 to 300 µm. In addition to H2B-Cherry, the following constructs were electroporated: in (A), LacZ, Mir30 shLuc (control); LacZ, Mir30 shPard3; Atoh1::Ntn1, Mir30 shPard3; Dcc at 0.25 µg, Mir30 shPard3; LacZ, Mir30 shJamC; Atoh1::Ntn1, Mir30 shJamC; and Dcc at 0.25 µg, Mir30 shJamC; in (B), LacZ, Mir30 shLuc (control); Pard3, Mir30 shLuc; Pard3, Mir30 shDcc; JamC, Mir30 shLuc; and JamC, Mir30 shDcc. Each respective control is represented by a red dashed-line in the distribution plot. (C) Chart representing the average variation across replicates in the endpoint nuclear displacement on the x-axis from an even probability of 50%:50%, with negative values representing an attraction to the source of the Ntn1 gradient (in red when statistically significant) and positive values representing repulsion (in blue when statistically significant). Unsorted dissociated CGNs were nucleofected and plated on laminin-coated channel microslides for 24 h, then Ntn1 was added unilaterally into the channel 10 min before the start of nuclear tracking for 2 h. Cells were nucleofected with H2B-mCherry, GPI-pHluorin, and the following: LacZ (control), Pard3, JamC, Mir30 shLuc (control), Mir30 shPard3, and Mir30 shJamC. Abbreviations: EGL, external granule layer; ML, molecular layer; IGL, internal granule layer. In (A) through (C), error bars represent the SEM. Statistics:. p ≤ 0.1, *p ≤ 0.05, **p ≤ 0.01, ***p ≤ 0.005, as assessed by an ANOVA followed by a Dunnett post hoc test against the respective controls in (A) and (B) and by a chi-square test in (C) against an even probability of 50%:50%. See also Table S1.

Next, we conducted epistasis experiments to test whether Pard3 and JamC influenced the effect of Ntn1 on GZ exit through Dcc in an ex vivo slice assay. Knockdown of both Pard3 and JamC was previously reported to prevent radial migration of CGNs and GZ exit [6] (Figure 5A). Overexpression of Ntn1 in the GZ or mild expression of Dcc stimulated GZ exit in Pard3-silenced cells, meaning that the loss of Pard3 was rescued by increasing the Ntn1 signaling (Figure 5A). In contrast, the phenotype observed with loss of JamC could not be rescued by overexpression of Ntn1, and mild overexpression of Dcc only restored GZ exit to the control level (Figure 5A). We then tested whether the knockdown phenotype of Dcc could be rescued by overexpression of Pard3 or JamC. Pard3 expression that promoted GZ exit also rescued knocked-down Dcc, restoring the level to that in the controls, and JamC expression rescued migration, restoring it to that seen in the control group (Figure 5B). In channel microslide assays, overexpression of both Pard3 and JamC in an unsorted population of dissociated CGNs plated on laminin led to a switch in the migratory response from attraction to repulsion to a source of Ntn1 (Figure 5C), whereas knockdown left the cellular migration unbiased to the Ntn1 source (Figure 5C). These results suggest that Pard3 and JamC contribute to how CGNs integrate Ntn1 signaling through Dcc, as their levels affect the outcome of cellular migration and are necessary for generating an oriented GZ repulsion migratory response (Figure 5C). This suggests that Pard3 and JamC synergize with Dcc to integrate the Ntn1 signal that guides CGNs out of the GZ as they differentiate. The ability of elevated Ntn1 levels to rescue the Pard3 phenotype implies that Dcc signaling deficits in Pard3-deficient cells can be compensated by boosting the Ntn1 signal. In addition, the upregulation of Dcc alone was sufficient to rescue the Pard3 LOF phenotype, further confirming its critical role in compensating for the loss of Pard3.

**Figure 6.**
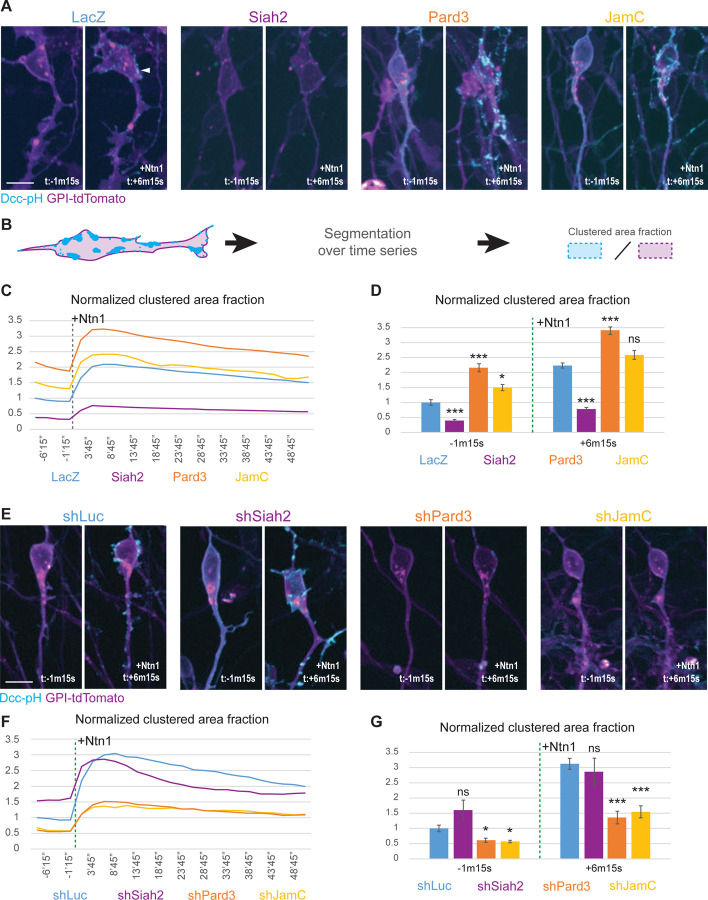
(A) and (E) Spinning-disk confocal live-cell imaging of dissociated granule neurons plated on laminin for 24 h after nucleofection with Dcc-pHlurorin (Dcc-pH) (cyan), GPI-TdTomato (magenta), and one of the following: in (A), LacZ (Ctrl), Siah2, Pard3 or JamC; in (E), Mir30 shLuc, Mir30 shSiah2, Mir30 shPard3, or Mir30 shJamC. Cells were tracked for a total of 1 h at 150-s intervals. Representative pictures for each condition show a maximum projection before (t: −1 m 15 s) and after (t: + 6 m 15 s) the addition of Ntn1 at 200 ng/mL. (B) Schematic representing the segmentation process for the analysis of the time-lapse images in (A) and (E). The Dcc-pH and GPI-tdTomato channels were segmented using Pixel classification with Ilastik. The resulting “clustered area fraction” is the ratio of the area of the segmented Dcc-pH regions (bright Dcc clusters) to the area of the membrane in each field of view for each time point. (C) and (F) Graphs representing the Dcc-pH area fraction over the membrane area, normalized to their respective controls. The different experimental conditions are the same as those in (A) and (E). A dashed-line marks the addition of 200 ng/mL of Ntn1 at t0. (D) and (G) Bar charts highlighting data presented in (C) and (F) for a time point before the addition of Ntn1 (t = −1 m 15 s) and for another time point shortly thereafter (t = +6 m 15 s). In (D) and (G), error bars represent the SEM. Statistics: ns, non-significant, *p ≤ 0.05, **p ≤ 0.01, ***p ≤ 0.005, as assessed by an ANOVA followed by a Games–Howell post hoc test against the respective controls. See also Table S1.

To test the hypothesis that Dcc availability was regulated by the expression of Siah2 in GNPs or Pard3 and JamC in differentiated CGNs, we used Dcc-pH as a reporter for Dcc at the membrane surface and measured the clustering of Dcc in response to Ntn1 being added to the culture medium [29, 30]. The ratio of the segmented clustered area to the total cell area was calculated over time before and after the addition of Ntn1 (Figure 6B). In the controls cells expressing LacZ, bright Dcc clusters rapidly appeared on the cell surface (Figure 6A, arrowhead), resulting in the clustered area fraction increasing minutes after the addition of Ntn1 (Figure 6C, blue) before slowly returning to normal. Overexpression of Siah2 resulted in a substantial decrease in Dcc at the cell surface, whereas both JamC and Pard3 expression were associated with an increased baseline presence of Dcc at the cell surface (Figures 6C and 6D). Under all conditions, adding Ntn1 increased the clustered Dcc membrane fraction with Pard3 levels being significantly higher than in the controls (Figure 6D). Knockdown of Siah2 resulted in a slight increase in Dcc at the membrane at rest, although this was not significant, and the increased clustered area after Ntn1 addition appeared to decay faster than in the control (Figures 6E–6G). Knockdown of either Pard3 or JamC strongly reduced the amount of Dcc presented at the cell surface and the clustering triggered after the addition of Ntn1 (Figures 6E–6G). Interestingly, Pard3 overexpression could rescue the knockdown of JamC phenotype on membrane-exposed Dcc at rest and after Ntn1 addition (Figure S5A-5B), suggesting that Pard3 and JamC might act through additional pathways to promote Dcc membrane recruitment or stabilization. JamC specifically appears to have a role in spatially anchoring Dcc clusters, as they see their mobility reduced (Blue arrowheads, Figure S5C), which is disrupted with the expression of the dominant negative JamC cytoplasmic domain protein (Movie S3) [31]. Moreover, JamC dominant negative construct expression also reduces the Dcc-pH signal in Dcc puncta after Ntn1 addition. Overall, these results indicate that the amount of Dcc presented at the cell surface is influenced by the cell polarity pathway and the JamC adhesion molecule.

Guided by questions about whether Dcc clustering could be affected by differentiation status, we expanded our studies to investigate the surface distribution of endogenous Dcc in CGNs through their differentiation process using live immunocytochemistry on sorted populations from dissociated CGNs from Atoh1-GFP knock-in mice (Figure S6A-6B). Using random forest 3D segmentation, the total volume of Dcc puncta at the membrane surface was measured for individual cells, then sorted by their Atoh1 Nuclear GFP intensity as a differentiation status indicator. The average Dcc puncta/cluster size population distribution was also assessed. Results show that the total membrane Dcc amount increases as progenitor (HH) differentiates into neurons (HM and HL) and eventually decreases as neurons continue to differentiate (HN and LN). Progenitors exhibit diffuse and smaller Dcc structures, while differentiated neurons have bigger aggregates localized more specifically at branching points and neurite tips (Figure S6A-6B). Consistent with our Dcc-pHluorin experiments, Pard3 and JamC expression increased endogenous Dcc at the cell surface (Figure S6D-F).

## Discussion

In the developing nervous system, progenitor GZ occupancy and subsequent postmitotic neuron migration are traditionally viewed as distinct neuronal maturation phases. Failing to recognize the coordination between these stages and the differential impact of extrinsic cues at specific developmental time points may cause us to overlook critical mechanisms that regulate the timing and sequence of maturation within a lineage, resulting in an incomplete understanding of neural development. While GZ exit and radial migration mark the transition from progenitor proliferation to terminal neuronal differentiation, how ‘station keeping’ between cells in these two states is communicated and interpreted at the cell biological level remains unknown. For example, increased cell adhesion can sort cells that share complementary profiles between layers in a developing nervous system tissue by increased cell adhesion molecule expression; however, this sorting is a deterministic event that requires little to no communication between the cells in each layer. In this study, we used the GZ exit of differentiating CGNs of the cerebellum as a model to study how extracellular guidance cue receptors, cellular polarity complexes, and adhesion proteins can intersect, creating a coincidence detection circuit that modulates cell migration behaviors.

We found that Ntn1 signaling affects the somal translocation direction of CGNs. Interestingly, we observed a gradual shift in Ntn1-dependent migratory behaviors; GNPs are unresponsive to this signal, which ultimately transitions to one, causing CGNs to be repulsed by a source of Ntn1 as these cells terminally differentiate and mature. Although Ntn1 has previously been described as having a repulsive effect on the migratory behavior of postmitotic neurons [32, 33], this is the first time that a transitional behavior has been described for the same lineage based on the differentiation status of the cells and a change of substrate. It is critical to note that GNPs themselves produce the Ntn1 protein repulsive to the CGN progeny of this progenitor population. Thus, the unresponsiveness of GNPs to the Ntn1 signal from the GZ, while CGNs respond to it, creates the critical station-keeping event that separates these cells in the inner and outer EGL via differential migratory behaviors. Interestingly, most neuronal GZ cells in the developing nervous system express Ntn1 during their peak of proliferation, like what is seen in the ventricular zone of the spinal cord and the ganglionic eminence [33, 34]. This suggests that acquiring Ntn1 directional sensitivity by newborn neurons as they differentiate could be a common mechanism to prevent the cells from migrating or projecting back into other GZs [32, 35].

How is Ntn1 sensitivity ensured during CGN differentiation? We uncovered a coincidence detection circuit whereby JamC and Pard3 are required for Ntn1-induced GZ exit because of the ability of these proteins to increase basal levels of the Dcc receptor at the plasma membrane and to increase Ntn1-induced Dcc exocytosis. Although many signaling pathways have been characterized as transducing Dcc signaling in response to Ntn1, this new mechanism ultimately controls the level of CGN repulsion via Ntn1 by affecting the number and distribution of signal receptors at the cell surface. Previous results from our laboratory have shown that Pard3 causes JamC exocytosis only in differentiated CGNs, which is required for GZ exit. In this study, we have expanded on these results to show that Dcc and JamC extracellular domains interact. Moreover, super-resolution Airyscan microscopy revealed that a subset of Dcc receptors localizes to the periphery of JamC adhesion plaques and that Dcc exocytosis after Ntn1 addition occurs near JamC adhesion plaques. Thus, we have provided functional evidence that Pard3 and JamC cooperate with Dcc to promote GZ exit, and the effect of adding Dcc at JamC adhesions suggests a coincidence detection mechanism whereby engaged adhesion receptors at the cell surface provide a template for adding more Dcc. The polarity protein Pard3 also positively affects the amount of Dcc at the cell surface. However, it is unclear whether this results from a direct action promoting Dcc exocytosis and/or stabilization of large Dcc aggregates at the membrane surface. The connection between Dcc and the Pard3–JamC polarity-dependent adhesion pathway that creates neuronal adhesion during GZ exit is intriguing. Dcc has been noted to create cell polarity events, including effects on axon formation, and directed invasions of cells in development [25, 35–37]; however, no connection between Dcc and the core polarity pathways has previously been described. Cell migrations frequently require oscillatory mechanisms in cytoskeletal organization or receptor recruitment that are stabilized by cell polarity pathways [36, 38]. The coincidence detection circuit between Pard complexes and JamC adhesions promotes such oscillatory stabilization: In unmanipulated CGNs, Dcc receptors are quickly cleared from the cell surface after bath application of Ntn1, whereas the clearance of Dcc receptors is enhanced by Pard3/JamC loss of function and delayed by Pard3/JamC gain of function. Differentials of Dcc receptors stabilization and subsequent activity at the cell surface coincident with Pard3/JamC localization promote CGNs cell bodies to exit its GZ, which is essentially a directionally polarized behavior. It will be of great interest to understand how this GZ circuit is precisely controlled at the subcellular level: Although the CGN soma migrates in an Ntn1/Dcc-dependent fashion towards the IGL, CGN parallel fiber axons and their growth cones maintain their position in the molecular layer even in the presence of Ntn1.

While Ntn1 response is often studied in the growth cone of developing neurons, we also demonstrate that control of Dcc levels and access to the membrane is key to regulating the exit of CGN soma from the EGL. Here, we have confirmed that Dcc is a Siah2 target and have shown that Ntn1-dependent GZ exit can be restrained in GNPs through targeted proteasomal degradation of Dcc by Siah2. Interestingly, Siah2 frequently targets multiple components of particular cell biological processes for degradation [23]. This appears to be the case for the Ntn1– Dcc GZ exit pathway, as Siah2 also targets Pard3 for degradation, which positively regulates Dcc membrane occupancy. Siah2 expression in GNPs can restrain Ntn1-induced GZ exit by directly targeting Dcc or factors that promote Dcc membrane occupancy. By equally promoting Pard3 degradation and, therefore, inhibiting JamC access to the cell surface [6], Siah2 acts as a master regulator of Ntn1 sensitivity in GNPs. Determining whether Siah2 has further roles in modulating Dcc will be interesting, as previous studies have shown that Ntn1 can promote Dcc ubiquitination [39]. Indeed, given the rapid disappearance of surface Dcc after Ntn1 addition, Siah2 activity may be regulated by Ntn1 activity, or Siah2 affinity for Dcc may be modified due to Ntn1 binding. It is worth noting that another ubiquitin ligase, Trim9, also regulates Dcc clustering. Trim9 loss of function compromises Dcc clustering, but the loss of clustering is related to unrestrained exocytosis of a host of cargos controlled by soluble N-ethylmaleimide attachment protein receptor (SNARE) proteins due to focal adhesion kinase activation [40]. Interestingly, Siah2 degrades Trim9; it may be interesting to dissect whether this additional ubiquitin ligase is part of a larger ubiquitin ligase circuit regulating Dcc signaling in cooperation with Siah2.

We demonstrate that Unc5c, a prototypical Dcc co-receptor, is necessary and sufficient for CGN GZ exit. While Unc5 homologs are traditionally known to convert Dcc-Ntn1 responses from attraction to repulsion, particularly in growth cones [14], their role in neuronal migration in mouse models is more nuanced. Earlier studies suggested that Unc5 homologs negatively regulate neuronal migration. For example, increased Unc5b expression in Sip1 mutants reduces cortical interneuron migration into the developing cerebral cortex [41]. Yamagishi et al. and Seiradake et al. also showed that downregulation of Unc5d, in response to FLRT receptors, is required for cortical neurons to leave the intermediate zone and move to the cortical plate, while elevated Unc5d expression slows this migration [42, 43]. In contrast, Akkermans et al. proposed that newborn cortical neurons are repelled from ventricular zone radial glia due to a trans-recognition of Glypican-3 and Unc5d, which is partly analogous to the oEGL repulsion we report here [44]. Our finding that elevated Unc5c expression converts attraction to repulsion aligns with the classical role of this co-receptor pair. However, the differentiation-specific expression of Unc5c introduces a new concept: differentiation status and the continuum of differentiation states can significantly alter a cell lineage’s Ntn1 responsiveness. In this case, a complex interplay of polarity proteins, adhesion molecules, and Unc5c. How does Pard3 or JamC GOF promote repulsion? Our model that involves receptor stabilization and differentiation status inputs suggests two interpretations that are not mutually exclusive. In the first simple scenario, increased Pard3 and JamC can increase Dcc surface occupancy in maturing CGNs already experiencing higher Unc5c expression levels, thus promoting repulsion. Indeed, our observation that a mild elevation in Dcc induces repulsion supports the notion that higher surface Dcc is sufficient to trigger this response in cerebellar slices. In the second scenario, it’s also possible that Pard3 GOF can promote CGN differentiation directly, which could favor the CGN-differentiation-specific expression of Unc5c. Interestingly, a recent modeling study by Limerick et al. suggested that C. elegans Unc5 unexpectedly regulates asymmetric Unc40 localization, the worm Dcc ortholog; however, no connections were made in this model to classic polarity proteins [45]. Our results suggest that given Pard proteins cooperate with Dcc, an ancient polarity-inducing receptor, the cerebellar system may also provide an opportunity to uncover Pard-Unc5 connections related to Dcc clustering and membrane asymmetry.

The differential responsiveness of the CGN lineage to Ntn1 contrasts sharply with other contexts where cell-intrinsic states modulate Ntn1 responses. For instance, Moo-Ming Poo’s lab showed that calcium transients from non-selective cation channels can rapidly alter nerve growth cone responses to Ntn1, independent of fate changes [46]. Similarly, in spinal cord motor axons, Unc5c and EphrinB2 create an integrative guidance response between Ntn1 and ephrins, guiding medial LMC axons [47]. This synergy may be driven by differential transcriptional gene expression during lateral or medial LMC specification [48], yet differentiation status was not assessed. Our work uncovers a distinct mechanism by which differentiating neurons exit the GZ, emphasizing the role of cell polarity in modulating the interaction between adhesive signals and Netrin-1. Unlike models focused on rapid growth cone behavior changes or fate-defined axon guidance, our work reveals how cell polarity and adhesion proteins create a coordinated circuit that regulates Netrin-1 receptor availability. This circuit effectively gates the repulsion needed for GZ exit and initiates radial migration through differentiation-driven changes in cell biology, impacting surface levels of Dcc. The increased expression of Unc5c and Pard3 during the GNP to CGN transition, coupled with the more effective JamC adhesion in CGNs that regulates Ntn1 responsiveness through Dcc, highlights this transition as a pivotal step toward terminal differentiation. Siah-mediated degradation of Pard3 and Dcc in GNPs illustrates how the progenitor state actively represses these CGN-specific events, reinforcing the differentiation switch in Ntn1 sensitivity and the coincidence detection circuit. While Dcc, an ancient polarity-inducing molecule, has been studied extensively, our work is the first to connect Dcc to a classical cell polarity signaling cascade, specifically linking it to Pard3. This connection integrates Dcc into established polarity mechanisms, revealing how polarity signaling drives terminal differentiation. It allows differentiating neurons to navigate complex niche environments, effectively aligning their trajectory with a defined differentiation path while overcoming the challenges of integrating multiple extrinsic cues during germinal zone exit.

## Methods

### Mice

All mouse lines were maintained in standard conditions (e.g., pathogen-free and with continuous access to food/water) in accordance with guidelines established and approved by the Institutional Animal Care and Use Committee at St. Jude Children’s Research Hospital (Protocol no. 483). B6.129(SJL)-Ntn1tm1.1Tek/J, Tg(Atoh1-cre/Esr1*)14Fsh/J, B6.Cg-Gt(ROSA)26Sortm9(CAG-tdTomato)Hze/J, B6.129S-Atoh1tm4.1Hzo/J, and C57BL/6J mouse strains were obtained from The Jackson Laboratory. Neonates were collected on postnatal days 7 through 9 for the studies detailed in the Methods section below or as indicated in the experimental details in the figures. Male and female mice were mixed for the described experiments as no effect of sex on the timing of GZ exit or migration initiation has been observed so far in scientific literature.

### Method Details

Blinding was not used in data collection.

### Plasmid vectors

Expression plasmids for LacZ, Pard3a, Siah2, Siah2ΔRING, and H2B-mCherry were subcloned as previously described [6, 7] pCIG2 Dcc-pHluorin (Dcc-pH), with a pH-sensitive form of green fluorescent protein (GFP) [49] inserted in position 1090, was obtained from Franck Polleux at Columbia University. All new cDNA constructs encoded mouse (*mus musculus*) proteins and were cloned in the laboratory by the overlapping PCR method. See the Key Resource Table for a complete recombinant DNA list.

### Western blot analysis

HEK293T cells were lipofected (using Lipofectamin 2000; Thermo Fisher Scientific) 1 day before being harvested. Cells were lysed using Lysis/Binding/Wash Buffer (Cell Signaling Technologies) with Halt^™^ Protease and Phosphatase Inhibitor Cocktail (Thermo Fisher Scientific) and reduced using Laemmli buffer (Sigma-Aldrich, cat. no. S3401). Samples were subjected to polyacrylamide gel electrophoresis using the Bolt^™^ system (Thermo Fisher Scientific) and transferred to PVDF membranes (Immobilon^®^-FL PVDF Membrane), blocked in Intercept blocking Buffer, and immunoblotted with appropriate antibodies (see the Key Resource Table for dilutions). For immunoprecipitation and co-immunoprecipitation experiments, the Protein G Immunoprecipitation Kit was used with 1 µg of anti-GFP antibody (RRID: AB_221569) or control IgG (RRID: AB_2722735) per the manufacturer’s protocol. Samples were then blotted as previously described. HEK293T cells used for blot analysis of the K48-linked ubiquitin signal were treated for 6 h with the proteasome and calpain inhibitor MG132 (50 µM) before being subjected to lysis to see the ubiquitin mark.

### Ex vivo cerebellar electroporation, organotypic slice culture imaging.

Cerebella of P7 WT (C57BL/6J) mice were dissected, and the superficial layer of the meninges was removed. Cerebella were soaked in endotoxin-free plasmid DNA suspended in Hank’s balanced salt solution (1–3 μg/μL of each DNA was generally used, with pCIG2-mCherryH2B being electroporated as a nuclear marker for migrating CGNs), transferred to a CUY520-P5 platinum-block Petri dish electrode (Protech International), and electroporated with a CUY21EDIT square-wave electroporator (90 V, 5 pulses, 50-ms pulse, 500-ms interval) (Protech International). Electroporated cerebella were embedded in 4% low-melting-point agarose, and 300-μm sagittal cerebellar slices were prepared using a VT1200 Vibratome (Leica Microsystems). Slices were transferred to Millicell tissue culture inserts (Millipore) and incubated in serum-free medium (FluoroBrite^™^ DMEM supplemented with 2 mM L-glutamine, 50 cU/mL penicillin–streptomycin, and 1 × B27 and 1 × N2 supplements [Gibco]).

To measure the migration distance of CGNs, cerebellar slices were fixed with 4% paraformaldehyde, mounted on slides using ProLong Gold (Invitrogen), and imaged at 20× with a spinning-disk confocal microscope. Measurements were made with the Amira software, using a self-written script for detecting local maxima of H2B-mCherry–positive nuclei and a Euclidian transform giving the distance from the cerebellar pial surface. Statistical analysis were performed, and graphs were prepared with RStudio software.

### Spinning disk confocal microscope

Imaging was performed using a Marianas Spinning Disk confocal microscope (Intelligent Imaging Innovations), which includes a Zeiss Axio Observer microscope with the following objectives: ×40/1.0 NA Plan-Apochromat (oil immersion), ×63/1.4 NA Plan-Apochromat (oil immersion), and ×40 C-Apochromat 1.2 W Corr M27 (WD=0.14–0.28 mm). The system had an Ultraview CSUX1 confocal head and 440–514 nm or 488–561 nm excitation filters. An ImageEM-intensified CCD camera (Hamamatsu) was used for high-resolution imaging. Video recordings were captured using Slidebook software (Intelligent Imaging Innovations).

### Preparation and nucleofection of CGNs

Briefly, cerebella were dissected from the brains of P7 WT (C57BL/6J) mice, then the tissue was coarsely chopped and treated with a Neural Tissue Dissociation Kit (Miltenyi Biotec). The suspension was layered onto a discontinuous Percoll gradient (35% and 60%) and separated by centrifugation. Cells at the 35%–60% interface were isolated. The resulting cultures routinely contained >95% CGNs and <5% glia. Plasmid expression vectors encoding proteins of interest were introduced into granule neurons via Amaxa nucleofection, using an Amaxa Mouse Neuron Nucleofector Kit in accordance with the manufacturer’s instructions and program O-005. The concentration of pCIG2 expression vectors used for each construct (5 to 25 µg of DNA per 6 million cells) was determined empirically but was consistent across replicates. After cells had been allowed to recover from the nucleofection for 10 min, they were plated in appropriate culture chambers and maintained in culture for 24 h in serum-free medium. For analysis of the Dcc clustering response to Ntn1 (Figure 6), immunocytochemistry (Figures S2B and S4B), and live imaging (Figures 4A–4C; Figures S4C and S4D), we used 6-cm dishes with glass bottoms that had been treated with poly-L-ornithine then coated with laminin at 1 µg/cm^2^.

### Atoh1-eGFP–sorted cells

Mouse pups homozygous for Atoh1::GFP (B6.129S-Atoh1tm4.1Hzo/J; The Jackson Laboratory, JAX: 013593) were collected at P7, and cerebellar granule neurons were isolated after dissociation by the methods described above. “High” and “low” populations were sorted, based on their GFP intensity, with a cell sorter (FACSAria Fusion, BD Biosciences; 85-μm nozzle, 35 PSI). The GFP intensity distribution profile was constant (Figure S2A), and the gating criteria were consistent across replicates.

### Migration assays on channel microslides

For migration assays, microslides (µ-Slide I; Ibidi, cat. no. 80106) were treated with poly-L-ornithine and then coated with laminin at 1 µg/cm^2^ or vitronectin at 2 µg/cm^2^. Two million nucleofected CGNs or freshly sorted “low” or “high” Atoh1::GFP cells (prepared as described above) were then added to the channel and left to incubate. After 24 h, before the start of the time-lapse image acquisition, the medium was replaced twice by aspirating the existing medium from one end of the track and adding 100 µL of warm, fresh medium at the other end. After a 10 min incubation, the same procedure was repeated, but this time adding only 50 µL of serum-free medium containing Ntn1 to a final concentration of 200 ng/mL or the equivalent volume of 1× PBS as a control. Cells were then tracked every 150 s for 130 min after Ntn1 addition in a spinning-disk confocal microscope with environmental control. Cell body migration was tracked manually with SlideBook (Intelligent Imaging Innovations) by using the H2B-mcherry signal (in nucleofected cells) or differential interference contrast (DIC) (in Atoh1-sorted cells). Cells showing less than 10 µm of total displacement over the time-lapse period were analyzed.

### Cerebellar immunohistochemistry

Postnatal brains were fixed by immersion in 4% paraformaldehyde at 4°C overnight, washed five times in 1× PBS for 24 h, and then cryoprotected in PBS containing 30% sucrose. Histologic sagittal sections were cut on a cryostat and pre-blocked for 1 h in PBS with 0.2 M glycine, 0.1% Triton X-100, and 10% normal donkey serum. Sections were incubated overnight at 4°C with the primary antibodies (see the Key Resource Table for list of antibodies and dilutions). This was followed by incubation at room temperature for 1 h with the appropriate Alexa Fluor–labeled secondary antibody (Invitrogen; diluted 1:1000) before mounting. Images were acquired with a spinning-disk microscope using SlideBook software.

### Proximity labelling assay

Proximity labelling assay was carried out using Duolink^®^ system (Sigma-Aldrich), following the manufacturer instructions and recommended controls, on dissociated CGNs plated on laminin and cultured at 37ºC for 24h after fixation 2% PFA/0.5% Glutaraldehyde. Two pairs of primary antibodies were used: Rabbit against Dcc (ECD) (ab273570, Abcam) and Goat against JamC (ECD) (AF1213, R&D systems), and Mouse against Dcc (ICD) (A-1, Santa Cruz) and Rabbit against Pard3 (07–330, Sigma-Aldrich).

### Pulse-chase assay in Atoh1::CreERT2; Ntn1^flox/flox^

Tamoxifen in corn oil was injected intraperitoneally (at 100 mg/kg body weight) at P0, P1, and P2. For pulse-chase migration assays, mice were injected intraperitoneally with 50 mg/kg of EdU 48 h before tissue collection. EdU incorporation was assayed with the Click-iT assay (Invitrogen) according to the manufacturer’s instructions.

### Quantification and statistical analysis

Standard image processing and analysis were done with Amira (Thermo Fisher), SlideBook, or Fiji software. For cluster area analysis, pixels were classified with a random forest classifier, using Ilastik for sum projection after drift and Fiji for photobleach correction. Analyzed metrics of quantitated data are expressed as the mean ± SEM or as an adjusted ratio of clustering relative to the control average at the initial time point. The Student *t*-test was used to compare two groups. A one-way analysis of variance (ANOVA) was used for multiple comparisons with the Dunnett post hoc test against controls (for ex vivo slices) or with the Games–Howell post hoc test (for cluster area analysis). A non-parametric post hoc test was used for the cluster area analysis because the data did not satisfy the homogeneity of variance. The chi-square test was used in the channel microslide migration assay to test the results against an even probability (0.5) of the relative endpoint displacement on the axis of the length of the channel being either toward or away from the source of the gradient. In this assay, data were expressed graphically as the percentage variation from 50%, showing the mean ± SEM across replicates. All statistical test assumptions were verified when required by the test.

## Supplementary Material

Supplement 1

## Figures and Tables

**Figure 7.**
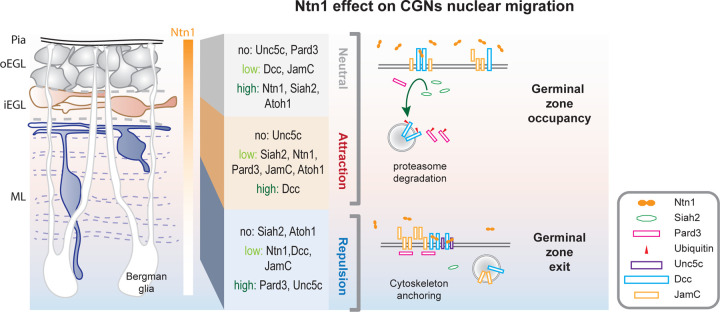
Model for the adhesion-guidance coincidence circuit. The left panel shows the various stages of CGNs and their relative layer occupancy (gray = GNPs, peach = newly differentiated CGN, blue = maturing CGN). The right panel shows the layer-specific response to Netrin-1 produced in the GZ. Siah2 degrades Dcc and Pard3 in GNPs rendering them unresponsive to Netrin-1. Newly differentiated CGNs are slightly attracted to Netrin-1, likely controlling how long these cells remain in the iEGL. Maturing CGNs express high levels of Pard3 which promotes exocytosis of JamC and Dcc and formation of larger structures at the membrane surface. The need of JamC adhesion for Dcc to exit the germinal zone in response to Netrin-1 is at the heart of the coincidence detection circuit.

## Data Availability

Scripting was done with Fiji and Amira. Original imaging data is available upon request.
