# Supplementary material for: Antagonistic action of Siah2 and Pard3/JamC to promote germinal zone exit of differentiated cerebellar granule neurons by modulating Ntn1 signaling via Dcc
Source: Res Sq. 2024 Sep 25:rs.3.rs-1819367. Preprint. [Version 1] doi: 10.21203/rs.3.rs-1819367/v1 (PMC11469462; doi:10.21203/rs.3.rs-1819367/v1)
Supplement: Supplement 1 [file NIHPPRS1819367V1-supplement-1.pdf]

Supplementary Materials

Additional methods

Quantitative Reverse Transcription Polymerase Chain Reaction

Total RNA was extracted from cultured CGNs using the RNAqueous™ Kit (AM1912, ThermoFisher), following the manufacturer's instructions. This was followed by a two-step real-time RT-PCR analysis on the ABI PRISM 7900 detection system. Random hexamers, TaqMan Reverse Transcription Reagents, and SYBR Green PCR Master Mix (Applied Biosystems) were used, as described by Singh et al. 2016 (Reference [7] in main text). Primer sequence: 18s Fwd: AGTAACGGAGCCAGACCTGAGA, 18s Rev: CTTTGAGGTGGAAGACACAGGAG, Siah2 Fwd: GGAACCTGGCTATGGAGAAG, Siah2 Rev: CAGGACAAGGACAGGAATAAGG

Live immunocytochemistry

Live immunocytochemistry was conducted to assess surface level of Dcc prior to fixation and permeabilization. Briefly, dissociated CGNs were nucleofected, and cultured on 16-well coverslip (Lab-Tek® chamber slide 16-well, 178599PK, Thermo-Fisher) coated with laminin (1 μg/cm^2) for 24h. After discarding media and wash in ice cold 1x HBSS, cells were incubated with the primary antibody (1/100 Goat anti Dcc AF844, R&D Systems) in 1x HBSS at 4ºC for 20min, wash twice in cold 1x HBSS, incubated with the secondary antibody (1/1000 Donkey anti-Goat A-21432, Thermo-Fisher) in 1x HBSS at 4ºC for 20min. After 3 washes in cold 1x HBSS, cells were then fixed in 2% PFA/0.5% glutaraldehyde at 4ºC for 10min, quenched in 0.2M Glycine in 1x PBS and mounted for imaging with Dapi.

Figure S1.

(A) (B) Sagittal cryo-sections through the cerebella of P9 Atoh1::CreERT2$^{+/-}$; Ntn1$^{flox/wt}$ or Ntn1$^{flox/flox}$ animals after three doses of tamoxifen (tm) at P0, P1, and P2. Sections were Immuno-stained against Cntn2 as a marker for the iEGL (A) and against Ki67 and Cleaved Caspase 3 (Cl-Casp3, as marker for proliferation and apoptosis respectively (B). Fraction of Ki67 positive over Dapi positive area in the EGL and the density of positive Cl-Casp3 cell in the EGL was quantified and charted for both phenotypes.

(C) Bar chart representing the average fraction of Ki67 positive nuclei over Dapi in dissociated CGNs plated on either Laminin, Vitronectin or Matrigel and cultured for 48 h with or without the addition of Netrin at 200ng/ml of media from t0. Cells were fixed after culture and immuno-stained against Ki67 and counter-stained with Dapi.

(D) Bar chart representing the average speed of the nuclei (in µm.s^{-1}) in dissociated cerebellar granule neurons plated on laminin for 24 h and tracked over 2 h at 150-s intervals. Cells were nucleofected with Centrin2-venus, H2B-mCherry, and Mir30 shRNA against luciferase (Luc, gray), Dcc (blue), or Unc5c (pink), and the results were separated based on cellular morphology. Cells with a progenitor-like morphology without clear polarity or a long neurite were categorized as multipolar, and cells with a defined polarity and a neurite longer than 30 µm were categorized as bipolar.

(E) Bar chart representing the average radial distance of nuclei of P7 mouse cerebellar cells electroporated in an *ex vivo* cerebellar slice assay, after 48 h in culture. The results show the effect of increasing concentrations of Dcc on germinal zone exit. Total DNA concentrations were adjusted to match the highest concentration of Dcc with the corresponding amount of LacZ.

Scale bars in (B) and (C) represent 20 µm. In (B-E), error bars represent the SEM. Statistics: n.s., non-significant, *$p \leq 0.05$, **$p \leq 0.01$, as assessed by a Student *t*-test in (B-D) and an ANOVA followed by a Dunnett post hoc test in (E). See also Table S1.

Figure S2.

(A) (Left) Immunohistochemical staining with an antibody against GFP in sagittal cryo-sections of Atoh1-GFP mouse cerebellum at P7, showing the restricted expression of Atoh1-GFP protein in the oEGL. (Right) Schematic representing the protocol used to isolate and sort the different cell populations from Atoh1-GFP mice cerebella. The GFP intensity profile shows three clusters, high, low, and negative (Neg), and the cells were separated accordingly.

(B) Immunocytochemical staining for Pax2, Pax6, and GFP in the different sorted Atoh1-GFP populations from (A).

(C) Density profile of the x-value for endpoint nuclear migration on channel microslides coated with laminin in response to an Ntn1 gradient for the LN population (black line) and the no-Ntn1 controls (red dashed-line) (see Figure 2E). Arrowheads indicate cells whose migratory behavior showed strong attraction (magenta) or repulsion (cyan) in response to Ntn1. Scale bars in (A) and (B) represent 50 µm and 10 µm, respectively. Abbreviations: oEGL, outer external granule layer; iEGL, inner external granule layer; EGL, external granule layer; ML, molecular layer; IGL, internal granule layer; GNPs, granule neuron progenitors; CGNs, cerebellar granule neurons; Neg, negative.

Figure S3.

(A) (Left) Immunohistochemistry staining of sagittal cryo-sections of Atoh1-GFP mouse cerebellum at P7, against GFP (Cyan) and Siah2 (Magenta), counterstained with Dapi (Gray). First graph shows the significative correlation between GFP and Siah2 signal intensity in individually segmented cell bodies. Second and third graphs show the boxplot distribution of Siah2 intensity (second) and the distance from the cerebellar surface (third) after binning each segmented nuclei in3 populations of granule neurons based on GFP intensity: “High”, “Low” and “Neg” (negative). (B) RT PCR against Siah2 on mRNA isolated from cerebellar granule neurons primary culture, with or without addition of mitogenic factor sonic hedgehog (Shh) after 24h of culture. The Graph represents the fold of Siah2 RNA over 18s RNA. (C) Sequence alignment of different Dcc orthologues, showing the conservation of the Siah degron motif, Pro-X-Ala-X-Val-X-Pro, within the intracellular P2 domain of Dcc. Scale bars in (A) represents 50 μm. In (B) error bars represent the SEM.

Figure S4.

(A) Western blot of lysates of HEK293T cells after lipofection with JamC-pHluorin (JamC-pH), Dcc, Dcc extracellular and transmembrane domain (Dcc-ECD), and Dcc intracellular domain (Dcc-ICD). Cell lysates were immunoprecipitated with an antibody against GFP (IP: GFP) or a negative-control rabbit IgG (Neg Ctrl IP). The blots were immunostained with two different antibodies against Dcc: one against the extracellular domain and one against the intracellular domain. Yellow arrowheads highlight the expected band sizes with Dcc, Dcc-ECD, and Dcc-ICD.

(B) Western blot of lysates of HEK293T cells after lipofection with Dcc-pHluorin (Dcc-pH), JamC-Halo, and Pard3-Halo. Cell lysates were immunoprecipitated with an antibody against GFP or a negative-control rabbit IgG. The blot was immunostained with an antibody against the Halo tag. The blot shows enrichment of JamC-Halo and Pard3-Halo in the eluate when Dcc-pH is pulled down with anti-GFP antibody.

(C) Confocal imaging and single focal plane images of a dissociated cerebellar granule neuron plated on laminin and maintained in culture for 24 h before fixation and immunohistochemical staining with antibodies against Dcc (cyan), JamC (yellow), and Pard3 (magenta). Magnifications of frames 1–3 are shown below the top image, with arrowheads highlighting areas where staining for Dcc and Pard3 (cyan and magenta arrowhead), Dcc and JamC (cyan and yellow arrowhead), Pard3 and JamC (yellow and magenta arrowhead), and Dcc, JamC, and Pard3 (white arrowhead) overlaps, or where staining for Dcc does not overlap with staining for other proteins (cyan arrowhead).

(D) and (E) Single focal plane image from Airyscan confocal live imaging of CGNs nucleofected with Dcc-pHluorin (cyan) and JamC-SNAP (yellow) constructs (D) or Dcc-pHluorin (cyan) and Halo-Pard3 (magenta) constructs (E). pHluorin and the SNAP dye used here are both pH sensitive, emitting fluorescence only if exposed to a neutral pH. Thus, they show only proteins currently at the cell membrane surface. The regions contained in the dotted rectangles are magnified below, showing where the Dcc-pH and JamC signal overlap at the membrane surface

at a point of contact between two neurites (D, white arrowheads) or where the Dcc-pH and cytoplasmic Pard3 signal overlap in the proximal dilation of a CGN (E, white arrowheads).

Scale bars in (C) represent 10 μm; those in (D) and (E) represent 5 μm.

Figure S5.

(A) Live Immunocytochemical staining for Dcc (Magenta) in dissociated CGNs from High and Low Atoh1-GFP (cyan) sorted populations (see Fig S2A). Cells were nucleofected with GPI-pHtomato (yellow) to visualize and segment the plasma membrane and counterstained with Dapi (white). Cells were plated at a low density and sorted by Atoh1-GFP intensity. High population was separated in 4 groups, H->High (progenitor-like with bigger nucleus) and H->Med (unipolar with smaller nucleus) compares to HH from Figure 3(A) here kept separated, H->Low, H->Neg and L->Neg compare to HL, HN and LN respectively. (B) Graph representing the average Dcc voxels segmented for individual cells for each developmental category from cells in (A). (C) Graph representing the average distribution of segmented objects per size in voxel for individual cells for each developmental category from cells in (A). (D) Dissociated cerebellar granule neurons nucleofected either with GPI-pHluorin (GPIpH, yellow) and LacZ as Control (Ctrl) or GPI-pHtomato (cyan) and 4 different conditions (Cond): LacZ, Pard3, JamC and Dcc. Both nucleofections (Ctrl) and (Cond) were mixed in 1:1 ratio, plated on laminin and culture overnight. Cells were then Live Immunostained for Dcc (magenta). (E) Cells cultured from (D) were segmented for both GPI markers and the LiveICC Dcc staining, showing an example of segmented masks. (F) Quantifications method related to (D) and (E). A segmented cluster to membrane voxel ratio was calculated for both “Ctrl” and “Cond”, and fold change was calculated for each image as a ratio of “Cond” over “Ctrl” ratios. The histogram displays the fold change for each condition. Scale bars in (A) (D) and (E) represent 10 μm. In (B) and (F), error bars represent the SEM. Statistics: *$p \leq 0.05$, **$p \leq 0.01$, as assessed by a Student *t*-test in (F). See also Table S1.

Figure S6.

(A) Graph complementing the results of Figures 6E–6G, representing the Dcc-pH area as a fraction of the total membrane area, normalized to the control. Here, there is an additional experimental condition, with CGNs being nucleofected with Pard3 and Mir30 shJamC. The dashed line marks the addition of 200 ng/mL of Ntn1 at t0.

(B) Bar chart highlighting data presented in (A) for a time point before the addition of Ntn1 (t = −1 m 15 s) and another one shortly thereafter (t = +6 m 15 s).

(C) Spinning-disk confocal live-cell imaging of dissociated granule neurons plated on laminin for 24 h after nucleofection with Dcc-pHlurorin (Dcc-pH) (cyan), GPI-TdTomato (Gray), and one of the following: LacZ (Ctrl), JamC or with a dominant-negative fragment of the JamC cytoplasmic domain (JamC DN) [31]. Cells were tracked for a total of 1 h at 150-s intervals. Representative pictures for each condition show a maximum projection before (t -1m15s) and +6m15s or +36m15s after the addition of Ntn1 at 200 ng/mL at t0. Arrowheads point at Dcc clusters.

(D) Graph complementing the results of (C), representing the Dcc-pH area as a fraction of the total membrane area, normalized to the control. The dashed line marks the addition of 200 ng/mL of Ntn1 at t0.

(E) Bar chart highlighting data presented in (C) for a time point before the addition of Ntn1 (t = −1m15 s), another one shortly (t = +6m15 s) and later (t= +36m15s) thereafter. The graph represents the average Dcc-pH intensity in either the membrane (non-cluster) or the segmented Dcc cluster area.

Scale bar in (C) represents 10 μm. In (B) and (E), error bars represent the SEM. Statistics: ns, non-significant, $^{*}p \leq 0.05$, $^{**}p \leq 0.01$, $^{***}p \leq 0.005$, as assessed by an ANOVA followed by a Games–Howell post hoc test against the control (shLuc) in (B) or Student t-test in (E). See also Table S1.

Supplemental Video Legends

MovieS1:

Single focal plane image from Airyscan confocal live imaging of CGNs nucleofected with Dcc-pHluorin (cyan) and JamC-SNAP (red) constructs. pHluorin and the SNAP dye used here are both pH sensitive, emitting fluorescence only if exposed to a neutral pH. Thus, they show only proteins currently at the cell membrane surface. This movie matches snapshot from Fig S4 D. Scale bar represents 5 µm; Time-stamp in sec.

MovieS2:

Max projection image from Airyscan confocal live imaging of CGNs nucleofected with Dcc-pHluorin (cyan) and Halo-Pard3 (magenta) constructs. pHluorin is pH sensitive, emitting fluorescence only if exposed to a neutral pH thus, showing only proteins currently at the cell membrane surface. This movie matches snapshot from Fig S4 E.

Scale bar represent 5 µm; Timestamp in min.

MovieS3:

Maximum projection of spinning-disk confocal live-cell imaging of dissociated granule neurons plated on laminin for 24 h after nucleofection with Dcc-pHlurorin (Dcc-pH) (cyan), GPI-TdTomato (Magenta), and one of the following: LacZ (Ctrl), JamC or with a dominant-negative fragment of the JamC cytoplasmic domain (JamC DN). Cells were tracked for a total of 1 h at 150-s intervals. This movie is complementary to Fig S5 C.

Ntn1 was added at 200 ng/mL at t0. Scale bar represents 10 µm; Timestamp in sec."

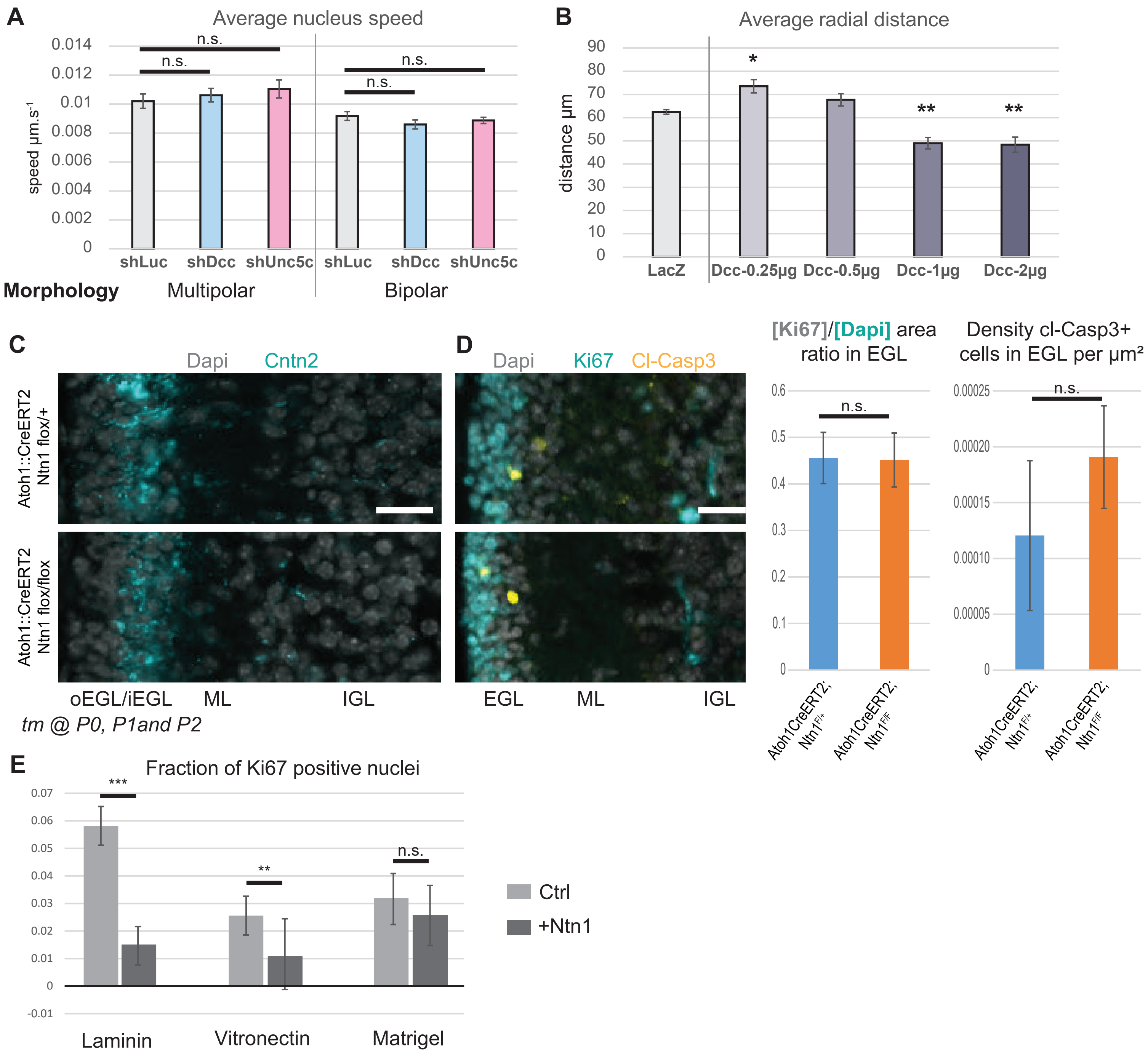
A
Average nucleus speed
speed μm.s-1
n.s.
shLuc
shDcc
shUnc5c
Morphology
Multipolar
Bipolar
B
Average radial distance
distance μm
LacZ
Dcc-0.25μg
Dcc-0.5μg
Dcc-1μg
Dcc-2μg
C
Dapi
Cntn2
Atoh1::CreERT2 Ntn1 flox/+
Atoh1::CreERT2 Ntn1 flox/flox
oEGL/iEGL
ML
IGL
tm @ P0, P1and P2
D
Dapi
Ki67
Cl-Casp3
EGL
ML
IGL
[Ki67]/[Dapi] area ratio in EGL
Density cl-Casp3+ cells in EGL per μm²
Atoh1CreERT2; Ntn1F/+
Atoh1CreERT2; Ntn1F/F
E
Fraction of Ki67 positive nuclei
Laminin
Vitronectin
Matrigel
Ctrl
+Ntn1

Figure S1.

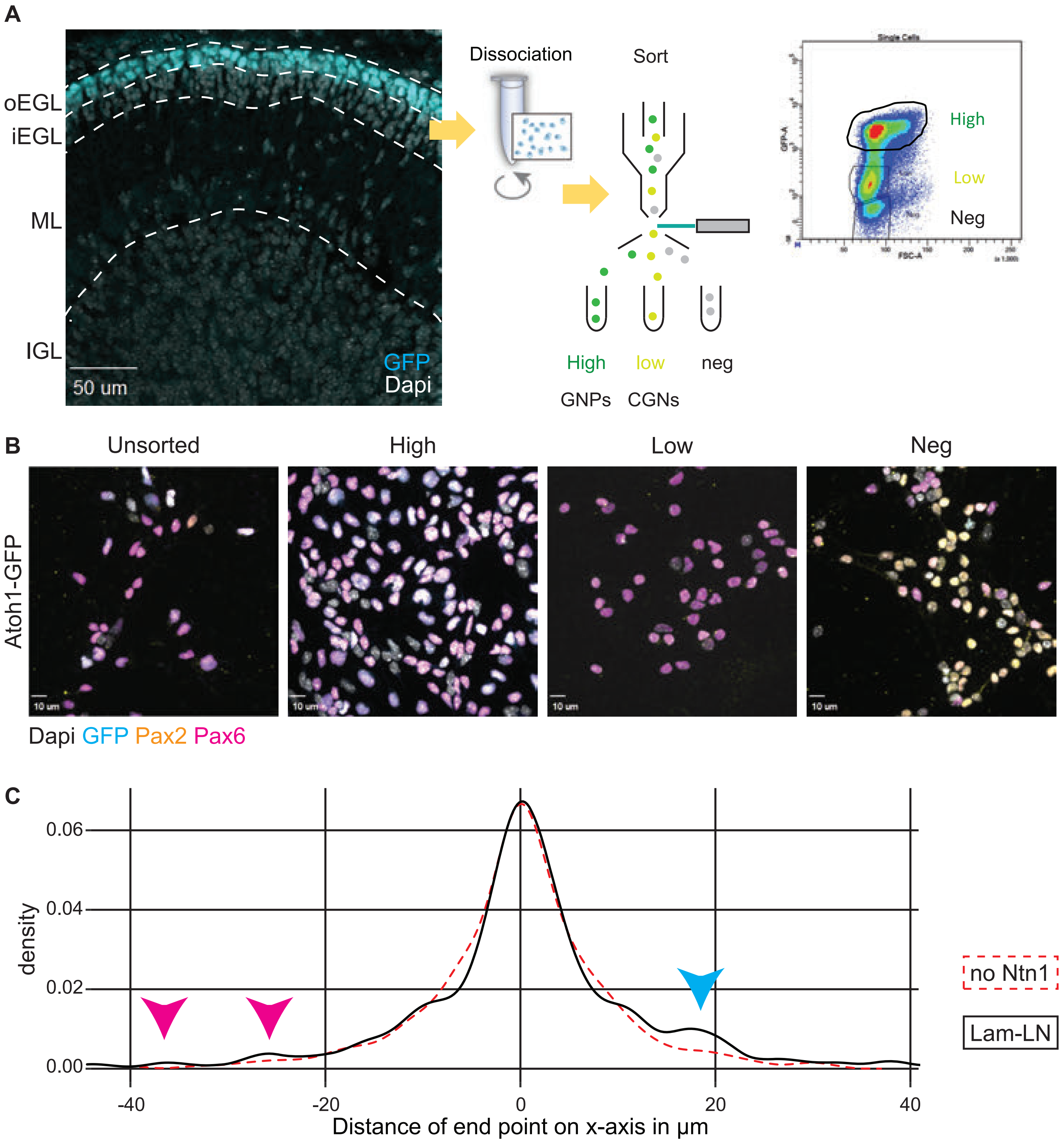
A
oEGL
iEGL
ML
IGL
50 um
GFP
Dapi
Dissociation
Sort
High
low
neg
GNPs
CGNs
High
Low
Neg
B
Unsorted
High
Low
Neg
Atoh1-GFP
10 um
Dapi GFP Pax2 Pax6
C
0.06
0.04
0.02
0.00
density
-40
-20
0
20
40
Distance of end point on x-axis in µm
no Ntn1
Lam-LN

Figure S2.

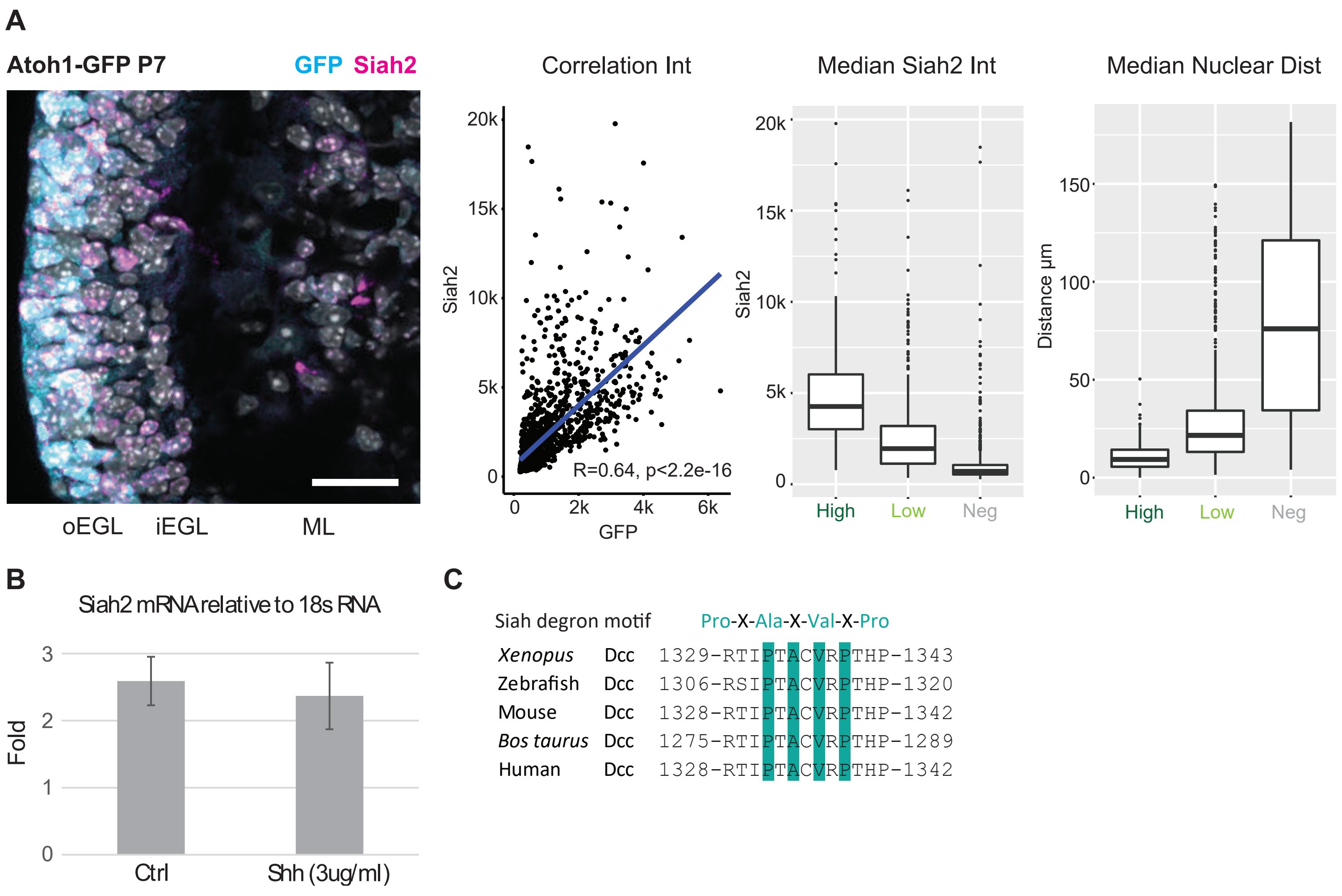
A
Atoh1-GFP P7
GFP Siah2
oEGL iEGL ML
Correlation Int
Siah2
GFP
R=0.64, p<2.2e-16
0 2k 4k 6k
0 5k 10k 15k 20k
Median Siah2 Int
Siah2
High Low Neg
Median Nuclear Dist
Distance μm
0 50 100 150
High Low Neg
B
Siah2 mRNA relative to 18s RNA
Fold
0 1 2 3
Ctrl
Shh (3ug/ml)
C
Siah degron motif Pro-X-Ala-X-Val-X-Pro
Xenopus Dcc 1329-RTIPTACVRPTHP-1343
Zebrafish Dcc 1306-RSIPTACVRPTHP-1320
Mouse Dcc 1328-RTIPTACVRPTHP-1342
Bos taurus Dcc 1275-RTIPTACVRPTHP-1289
Human Dcc 1328-RTIPTACVRPTHP-1342

Figure S3.

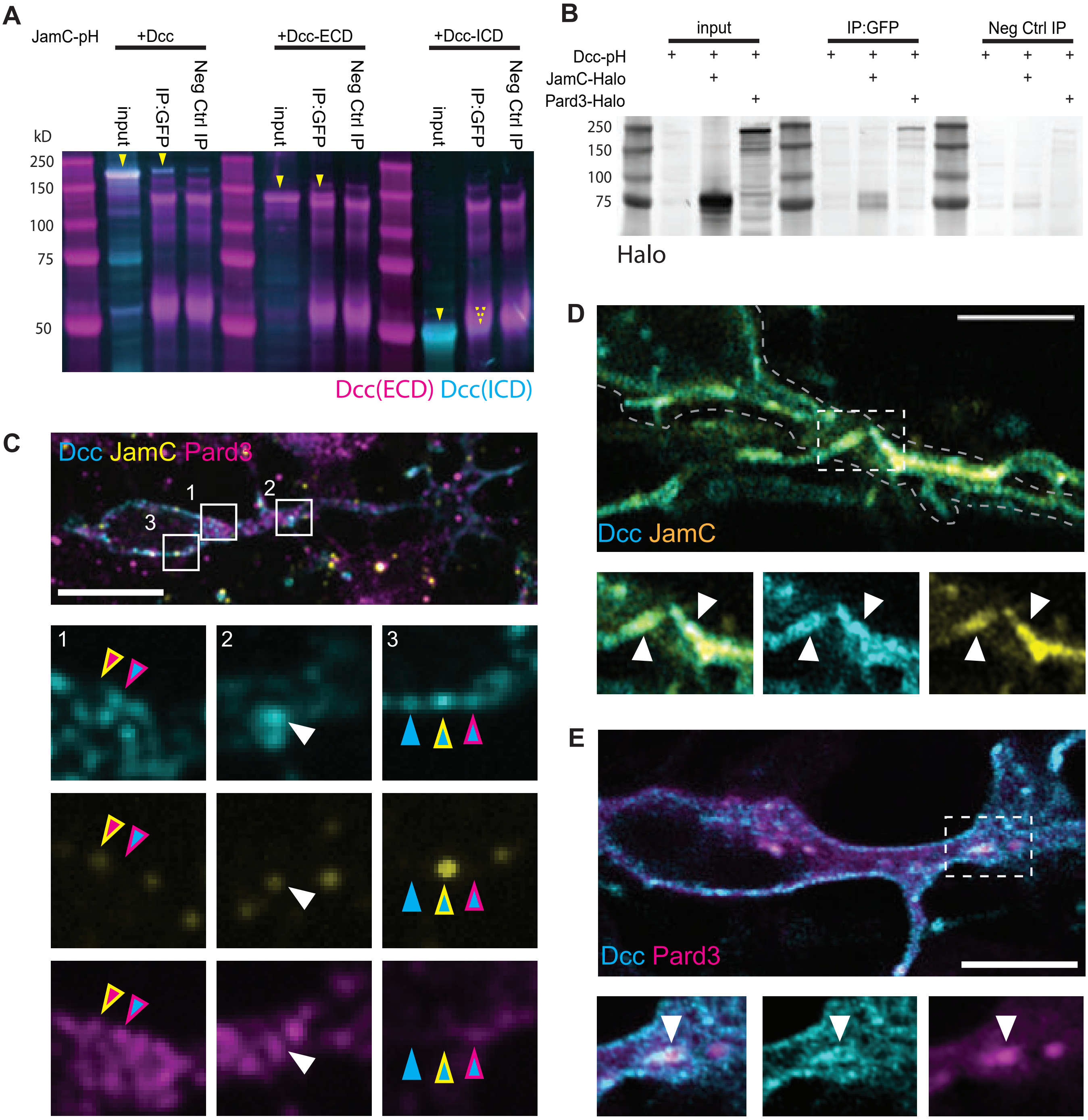
A
JamC-pH
+Dcc
+Dcc-ECD
+Dcc-ICD
input
IP:GFP
Neg Ctrl IP
kD
250
150
100
75
50
Dcc(ECD) Dcc(ICD)
B
input
IP:GFP
Neg Ctrl IP
Dcc-pH
JamC-Halo
Pard3-Halo
250
150
100
75
Halo
C
Dcc JamC Pard3
1
2
3
D
Dcc JamC
E
Dcc Pard3

Figure S4.

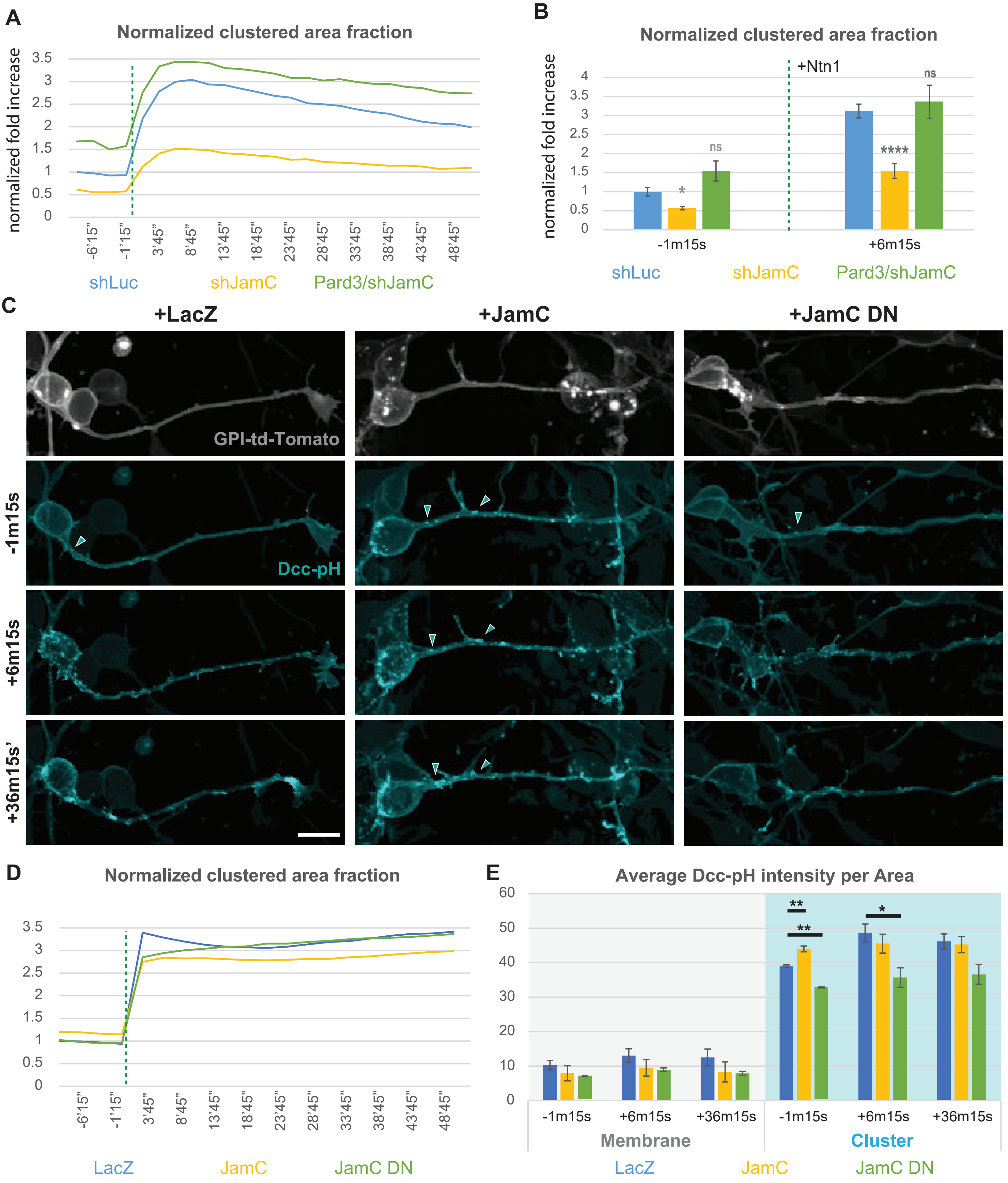
A
Normalized clustered area fraction
normalized fold increase
3.5
3
2.5
2
1.5
1
0.5
0
-6'15"
-1'15"
3'45"
8'45"
13'45"
18'45"
23'45"
28'45"
33'45"
38'45"
43'45"
48'45"
shLuc
shJamC
Pard3/shJamC
B
Normalized clustered area fraction
normalized fold increase
+Ntn1
4
3.5
3
2.5
2
1.5
1
0.5
0
*
ns
****
ns
-1m15s
+6m15s
shLuc
shJamC
Pard3/shJamC
C
+LacZ
+JamC
+JamC DN
GPI-td-Tomato
-1m15s
Dcc-pH
+6m15s
+36m15s'
D
Normalized clustered area fraction
3.5
3
2.5
2
1.5
1
0.5
0
-6'15"
-1'15"
3'45"
8'45"
13'45"
18'45"
23'45"
28'45"
33'45"
38'45"
43'45"
48'45"
LacZ
JamC
JamC DN
E
Average Dcc-pH intensity per Area
60
50
40
30
20
10
0
**
**
*
-1m15s
+6m15s
+36m15s
-1m15s
+6m15s
+36m15s
Membrane
Cluster
LacZ
JamC
JamC DN

Figure S5.

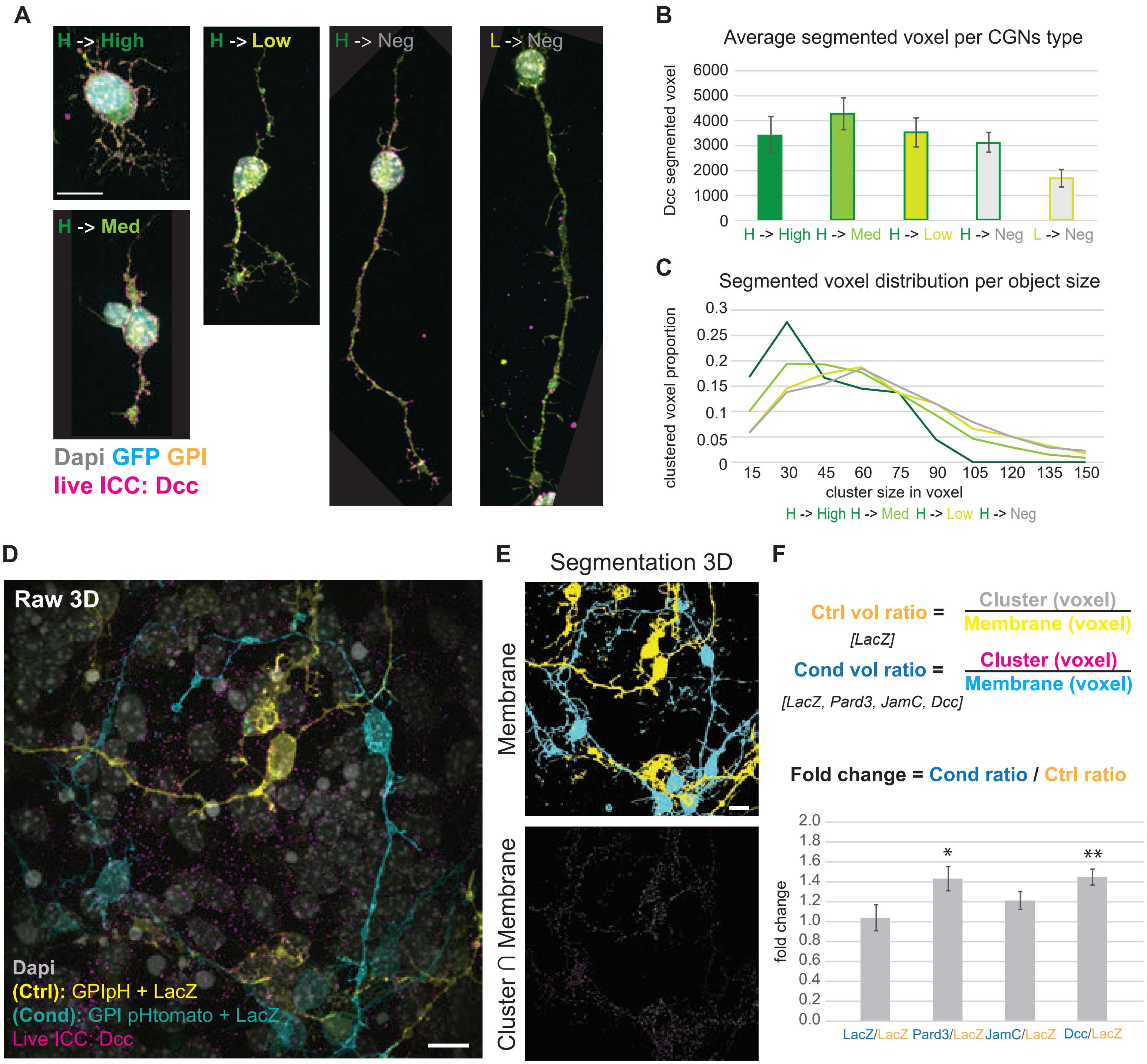

A
H -> High
H -> Low
H -> Neg
L -> Neg
H -> Med
Dapi GFP GPI
live ICC: Dcc
B
Average segmented voxel per CGNs type
Dcc segmented voxel
6000
5000
4000
3000
2000
1000
0
H -> High H -> Med H -> Low H -> Neg L -> Neg
C
Segmented voxel distribution per object size
clustered voxel proportion
0.3
0.25
0.2
0.15
0.1
0.05
0
15 30 45 60 75 90 105 120 135 150
cluster size in voxel
H -> High H -> Med H -> Low H -> Neg
D
Raw 3D
Dapi
(Ctrl): GPIpH + LacZ
(Cond): GPI pHtomato + LacZ
Live ICC: Dcc
E
Segmentation 3D
Membrane
Cluster ∩ Membrane
F
Ctrl vol ratio = Cluster (voxel) / Membrane (voxel)
[LacZ]
Cond vol ratio = Cluster (voxel) / Membrane (voxel)
[LacZ, Pard3, JamC, Dcc]
Fold change = Cond ratio / Ctrl ratio
fold change
2.0
1.8
1.6
1.4
1.2
1.0
0.8
0.6
0.4
0.2
0.0
*
**
LacZ/LacZ Pard3/LacZ JamC/LacZ Dcc/LacZ

Figure S6.

Supplementary Files

This is a list of supplementary files associated with this preprint. Click to download.

- 240804TableS1FiguresData.xlsx
- MovieS1JamCSnapDccpH.avi
- MovieS2DccpHHaloPard3.avi
- MovieS3.avi
